# Novel Trends in Hydrogel Development for Biomedical Applications: A Review

**DOI:** 10.3390/polym14153023

**Published:** 2022-07-26

**Authors:** Pablo Sánchez-Cid, Mercedes Jiménez-Rosado, Alberto Romero, Víctor Pérez-Puyana

**Affiliations:** Departamento de Ingeniería Química, Facultad de Química, Universidad de Sevilla, 41012 Sevilla, Spain; mjimenez42@us.es (M.J.-R.); vperez11@us.es (V.P.-P.)

**Keywords:** hydrogel, synthetic polymers, biopolymers, physical cross-linking, chemical cross-linking, composite hydrogels, biomedical applications

## Abstract

Nowadays, there are still numerous challenges for well-known biomedical applications, such as tissue engineering (TE), wound healing and controlled drug delivery, which must be faced and solved. Hydrogels have been proposed as excellent candidates for these applications, as they have promising properties for the mentioned applications, including biocompatibility, biodegradability, great absorption capacity and tunable mechanical properties. However, depending on the material or the manufacturing method, the resulting hydrogel may not be up to the specific task for which it is designed, thus there are different approaches proposed to enhance hydrogel performance for the requirements of the application in question. The main purpose of this review article was to summarize the most recent trends of hydrogel technology, going through the most used polymeric materials and the most popular hydrogel synthesis methods in recent years, including different strategies of enhancing hydrogels’ properties, such as cross-linking and the manufacture of composite hydrogels. In addition, the secondary objective of this review was to briefly discuss other novel applications of hydrogels that have been proposed in the past few years which have drawn a lot of attention.

## 1. Introduction

In recent years, humanity has witnessed exponential growth in different areas within the biomedical field, such as controlled wound healing, drug delivery and tissue engineering (TE), among others. For example, the demand and availability of tissues and organs for transplantation or regeneration of damaged tissues has become a significant issue [1]. TE is a multidisciplinary field that combines methods and principles of engineering and life sciences for the development of these biomaterials, which are excellent biological substitutes with the ability to maintain, restore or improve the function of target tissues [2]. Thus, TE relies on the design and development of scaffolds, which are highly porous 3D structures that can accelerate the regeneration of damaged tissues and organs by providing an optimal environment for cell growth and differentiation [3,4].

These scaffolds must be biocompatible and nontoxic to the organs and tissues in the body. They are usually loaded with cells, growth factors and drugs, as they can establish a synergistic combination, significantly improving cell retention rates in the local tissue; they also serve as carriers for growth factors and drugs. Moreover, scaffolds must also meet the mechanical requirements of the treated region once implanted [5,6,7]. Another requirement that must be met by scaffolds is their progressive biodegradation during the cell regeneration process while the new tissue is being formed [8]. The biodegradability of scaffolds allows avoiding the need to remove the material afterwards and prevents possible side effects that could arise from residues left in the body. Therefore, scaffolds must have some specific physicochemical and mechanical properties to achieve cell diffusion and 3D tissue formation [9].

Specifically, the extracellular matrix (ECM) has always been in the spotlight of many researchers in scaffold fabrication, due to its high biological compatibility and biodegradability. The ECM mainly consists of a combination of some proteins, polysaccharides and glycosaminoglycans. This complex mixture offers adequate biochemical and mechanical support to the surrounding cells, and it controls their performance in the regeneration process [9,10]. However, although TE has been considered a promising strategy to provide biological substitutes that can mimic the ECM, there are still important obstacles to overcome, such as the lack of specific materials for scaffold development and new procedures that enable the processing of delicate polymers such as proteins [11,12]. In this way, hydrogels have been extensively used in these biomedical research fields to overcome the main difficulties, as they can mimic many features of the native cellular microenvironment, due to their suitable properties for this purpose, e.g., their outstanding water retention capacity and flexibility [13,14,15,16].

Hydrogels are polymeric materials with a characteristic hydrophilic structure that enables the storage of large amounts of water and biological fluids in their 3D network. Hydrogels have interesting biomimetic properties, such as remarkable flexibility, softness, superior absorption capacity in their swollen state, nontoxicity, biocompatibility, biodegradability and tunable mechanical properties [17,18,19]. Due to these properties, hydrogels stand out for some specific biomedical applications compared to other nanomaterials (e.g., nanoparticles, nanofibers, thin films, etc.). Some advantages of hydrogels involve that they are usually biocompatible and capable of being injected in vivo as a liquid that gels at body temperature. In addition, they offer good transport of nutrients to cells and products from cells and their aqueous environment have the ability to protect cells and fragile drugs [20]. The reason behind the great water absorption capacity of these systems is the high content of functional hydrophilic groups contained in the polymer used as raw material. Moreover, their resistance to dissolution is due to the cross-linking structure generated between the polymer chains [16,21]. In addition, for hydrogel synthesis, the incorporation of a cross-linking agent is important to achieve a better structuring due to its ability to form new polymeric chains within the structure through a large variety of reactions between different polymeric molecules or fibrous proteins [22]. It is worth mentioning that any variation in monomer/polymer concentrations, structure or functionality, as well as the cross-linking agent used in the process, can significantly modify the structure, thus they must be adapted to the intrinsic features of the requested specific application [23,24].

Typically, hydrogels can be classified using two different categories, depending on the nature of the bond between the polymeric molecules that constitute the framework, namely: physically or chemically cross-linked hydrogels [25]. The manufacture method is another key factor for the development of each type of hydrogel system [26]. The structuring of physically cross-linked hydrogels mainly depends on weak and temporary interactions, such as molecular entanglements, ionic bonds, hydrophobic forces and hydrogen bonds, which are reversible and can be altered by environmental (physical) changes (e.g., temperature and pH) and do not produce any modification in the chemical structure [27,28,29]. Chemically cross-linked hydrogels are characterized by permanent and strong interactions between their polymeric chains, as they are connected by covalent bonds [30,31].

**Figure 1:**
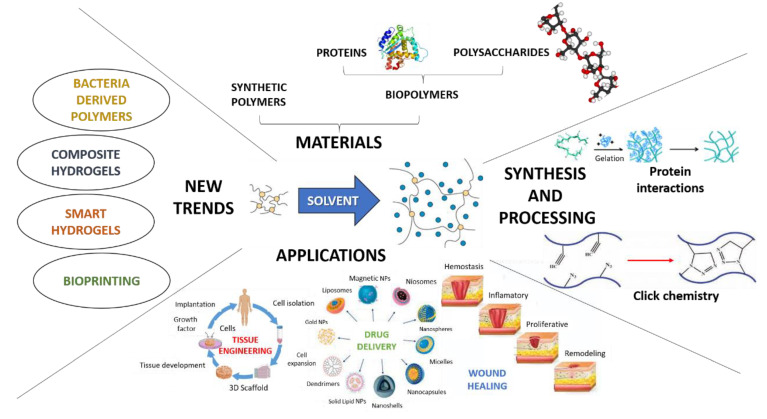
General perspective of the present and recent trends of hydrogels research in the biomedical field.

In addition to biomedical applications, hydrogels are also very appealing for many applications, such as self-assembly and catalysis, among others [32]. However, in this review article, the goal was to contextualize the current situation and challenges for hydrogel engineering for biomedical applications, as well as to compile the current trends in this field, with the premise that novel hydrogels with stronger and more stable properties are still needed and remain an important direction for research [33]. As was previously mentioned for TE, due to their properties and their ability to mimic ECM properties, hydrogels are suitable candidates for many biomedical applications, such as controlled drug release and wound healing, which are among the most well-known possible applications where hydrogels are posed as a promising solution [34,35,36,37]. Therefore, the objective of this review was to update and summarize current trends in applications and advances in hydrogel science, by means of the most used materials and synthesis methods for hydrogel manufacture, as well as different strengthening strategies, such as compositional modifications, different cross-linking techniques and hybridization. An overview of this purpose is depicted in Figure 1.

## 2. Materials

As was previously mentioned, hydrophilic polymers (natural and synthetic) are the most suitable materials for hydrogel manufacture. These polymers are characterized by containing polar functional groups, such as carboxyl, hydroxyl and amino groups, which make them swell (by water absorption), or water soluble [1,21,38]. The most used materials for the design and manufacture of hydrogels, along with their experimental stage and cell viability (to assess the biocompatibility of the systems based on these polymers) are summarized in Table 1.

Therefore, in this section, some of the most used materials for hydrogel fabrication will be described along with their advantages and drawbacks, as well as some of the current approaches proposed for each one in order to obtain novel hydrogels with stronger and more stable properties that may achieve eye-catching in vivo results.

### 2.1. Synthetic Polymers

Synthetic materials have very interesting properties for hydrogel applications in biomedicine, such as good controllability and excellent mechanical properties. However, the main problem with synthetic materials is that they lack the ability to induce the generation of new tissues quickly and effectively after being implanted, as well as the lack of antimicrobial activity [127]. Herein, the most used synthetic polymers will be described, presenting their advantages, drawbacks and their approaches to overcoming these challenges.

Poly (2-hydroxyethyl methacrylate) (pHEMA). pHEMA is a thermoplastic material that is not enzymatically degraded or hydrolyzed by acidic or alkaline solutions. pHEMA hydrogels are inexpensive, have excellent biocompatibility, non-biodegradability, high water content capacity, low thrombogenicity, cytocompatibility, abundant copolymer possibilities, soft materials with excellent temperature stability, acid and alkaline hydrolysis resistance, tunable mechanical properties and an optically transparent hydrophilic polymer that is desirable for various biomedical applications [39,40,128]. However, single pHEMA-based hydrogels have little commercial application; thus, numerous studies have been conducted to modify pHEMA structure with the aim of improving its properties, for example, using cross-linking agents such as ethylene glycol dimethacrylate (EGDMA) [41] and tetra(ethylene glycol) diacrylate (TEGDA) [42,43] to enhance its mechanical properties, or β-cyclodextrin-hyaluronan (β-CDcrHA) to reduce tear protein absorption in a contact lens [129]. Another approach is to copolymerize pHEMA with other polymers, commonly with polyacrylamide (PAA) [130] or ethylene glycol dimethacrylate (EGDMA) [40] to improve its mechanical properties, poly (ethylene glycole) diacrylate (PEGDA) to improve host biosensors or enhance its water absorption capacity [131], glycidyl methacrylate (GMA) [132] to facilitate cell attachment and proliferation, or 2-methacryloyloxyethyl phosphorylcholine (MPC) [133] to improve the water content retention and anti-biofouling properties. Moreover, other possibilities to improve pHEMA-based hydrogels’ performance for biomedical applications include the formation of composites, e.g., pHEMA with boric acid (BA), with interesting applications as soft contact lens material, as reported by Ulu et al. (2018) [134], or the formation of IPNs with gelatin to enhance biological properties for in vitro and in vivo performance [135]. Related to the biological properties of pHEMA-based hydrogels, Wang et al. (2022) reported the obtention of mechanically robust and biocompatible pHEMA-based hydrogels, combining pHEMA with maleic acid units (MA) and Fe^3+^, to suppress phase separation of pHEMA chains from aqueous solution and thus obtain a more homogeneous structure. These hydrogels were tested in vivo on 6 SD rats (12 weeks old), embedding samples subcutaneously and on porcine auricular cartilage replacement experiments. In addition, cell viability was evaluated by in vitro assays, obtaining results between 79–85% after 24 h cultures of each system, with no significant variations among them [44]. Kim et al. (2019) proposed the formation of a non-swellable and cytocompatible interpenetrated polymer network (IPN) between chemically cross-linked pHEMA and ionically cross-linked alginate, obtaining a synergistic effect that enhanced stiffness and toughness. Live/dead assay showed 99% cell viability over a period of 60 days [45].

Poly-vinyl alcohol (PVA). PVA is another interesting candidate for designing hydrogels for TE. It is a well-known hydrophilic polymer with remarkable biocompatibility [136]. Furthermore, PVA-based hydrogels are widely used in wound healing systems and ophthalmologic applications [137]. These are harmless, sterile and biocompatible, and they can absorb water efficiently [138,139]. However, it has been proved by Pan et al. that single PVA hydrogels do not efficiently support cell proliferation and lack antibacterial activity and elasticity [46]. Using cross-linking agents and combining PVA with natural polymers to improve their biological properties are current trends [46,47,48,49,138]. As to biological features, PVA-based hydrogels containing diphlorethohydroxycarmalol (DPHC) have been developed by Kim et al. (2020) for wound healing applications, whose in vitro and in vivo evaluation on ICR mice demonstrated interesting antimicrobial properties against *S. aureus* and *P. aeruginosa* [50]. On the other hand, Tummala et al. assessed the biocompatibility of PVA hydrogels reinforced with cellulose nanocrystals (CNCs) for ophthalmic applications, first on ex vivo porcine cornea [51] and subsequently on in vitro assays with human corneal cells [52], with promising results.

Poly (ethylene glycol) (PEG). PEG-based hydrogels have been promising candidates for biomedical applications in recent decades mainly due to their biocompatibility [53,140]. Moreover, their mechanical properties and chemical composition can be regulated, which allows easily controlling the architecture of the scaffolds, making PEG an interesting material for biomedical applications [141]. However, the main drawbacks of single PEG are its bio-inert nature, low antimicrobial abilities and severe volume expansion, thus it cannot provide a completely favorable environment to support cell adhesion and tissue formation, hindering its potential for biomedical applications [55,142]. Similar approaches such as pHEMA have been carried out to overcome these drawbacks and improve their performance for biomedical applications; for example, using cross-linking agents such as diacrylate [55] or pentaerythritol tetrakis (3-mercaptopropionate) (PETMP) [143], which enhance its mechanical properties. Nevertheless, the most recurrent strategy is copolymerization. There are several studies of PEG copolymerized with poly-vinyl pyrrolidone (PVP) [144], poly (ε-caprolactone) (PCL) [56,57], PLA [145,146] or different cellulose derivatives [147,148,149] to improve their performance for various biomedical applications. In this sense, Chen et al. (2022) developed an injectable PEG hydrogel with integrated lysozyme fibrils that implemented antiswelling and antibacterial capacities. In vitro tests with L929 cells and blood revealed that cell viability slightly decreased from 110–120% on the first day of culture to approximately 100–115% on the third day. In addition, in vivo tests with SD rats were carried out with promising results, posing this PEG-based hydrogel as an excellent candidate for biomedical applications [54]. In fact, Zhang et al. (2022) used this biomaterial for ophthalmic applications, testing it in vitro with corneal epithelial cells and ex vivo with cadaveric porcine eyes, resulting in promising candidates with remarkable wound healing properties after eyes surgeries [150]. In this line, Liu et al. (2021) evaluated the antibacterial performance of several injectable PEG-based hydrogels for promoting wound healing. In vitro assays with HaCAT cells showed that cell viability was maintained in the range of 80–100% for three weeks, reaching approximately 85% in the first week, subsequently increasing to 100% after the second week and lastly decreasing to 80%. Additionally, in vivo (SD rats) results revealed that these PEG-based hydrogels were able to promote wound healing whether gentamicin (a common antibiotic) was loaded or not [58].

Poly (N-isopropylacrylamide) (pNIPAAm). This polymer is the most investigated thermo-sensitive polymer [59]. According to the literature, pNIPAAm has an approximate 32 °C lower critical solution temperature (LCST) in water. This means that this polymer in solid state could dissolve into the solution once the temperature decreases below the LCST, exhibiting a thermo-reversible phase transition. Thus, copolymers derived from pNIPAAm have drawn massive attention in the study of the so-called “smart” materials or “smart” hydrogels for various biomedical applications, such as drug and gene delivery, enzyme and cell immobilization or biosensors [60]. Christiani et al. (2021) embedded alginate microparticles to tune the properties of an in situ formed hydrogel of a pNIPAAm copolymer for intervertebral disc replacement and repair. This formulation enhanced initial injectability, mechanical performance and bioadhesive interactions. In addition, cell viability calculated from in vitro assays of human adipose-derived mesenchymal stem cells (ADSMSCs) was obtained in the range of 90–100% with and without microparticles embedded. In vivo experiments were carried out on ex vivo porcine lumbar spines (5–6 months) with composite hydrogels, offering excellent results, as composites were able to resist expulsion under tension-compression and lateral bending when implanted [61].

Poly (N-vinylcaprolactam) (PNVCL). PNVCL is a biocompatible thermo-responsive synthetic polymer. It is a temperature-sensitive polymer with a LCST around 33 °C in water. Furthermore, lineal and/or branched PNVCL-based polymers are more susceptible to non-solubilization. On the other hand, cross-linked polymers go from a hydrated to a dehydrated state. It is worth mentioning that the LCST of PNVCL can be precisely tuned up and down by copolymerization with other monomers, such as N-vinylpyrrolidone [62,151]. Despite the difficulty of controlling the polymerization and copolymerization of PNVCL, compared with other thermo-sensitive polymers, PNVCL-based polymers have gained considerable attention in several biomedical areas, such as drug delivery systems, antibiotics, TE, and diagnostics and imaging, due to their biocompatibility, thermo-responsiveness and water-soluble behavior below the LCST [62,63,64]. However, there is not much information on the biological characterization of PNVCL-based hydrogels. Durkut et al. (2020) characterized in vitro performance of a PNVCL-g-Alg-NH_2_ copolymer with cytotoxicity and hemocompatibility assays, culturing human dermal fibroblast. Cell viability increased from ~85% to approximately 95% when aminated alginate proportion was diluted from 1/1 to 1/4 [65]. Further research is necessary to carry out in vivo investigation.

**Figure 2:**
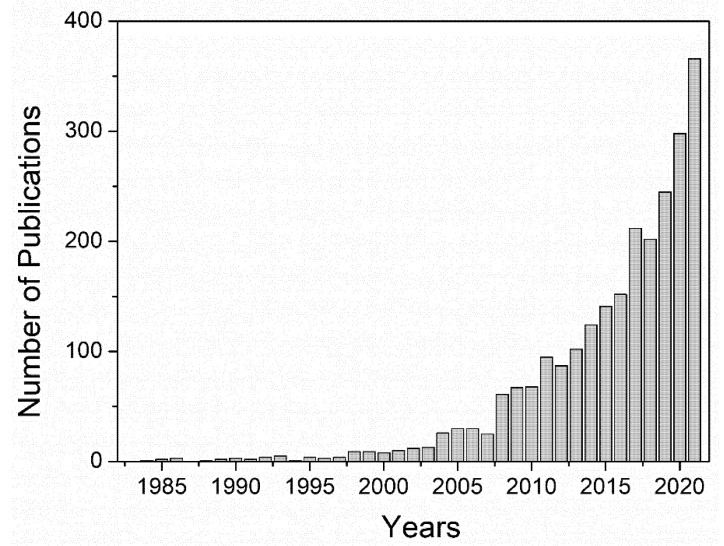
Evolution of the number of publications related to “natural polymers” and “hydrogels”. Data obtained from Scopus.

In conclusion, synthetic polymers have been widely used for decades due to their remarkable mechanical properties and their interesting capacity to modify their structure to improve their biomedical performance. The main drawback of these materials is that even though some of these polymers are biocompatible, such as PVA and PEG, synthetic polymers are still characterized by their limited biological properties once implanted in the human body. To improve these features, for example, cell viability, as shown in Table 1, the main approach is to combine synthetic polymers with natural polymers or molecules that can improve cell viability or other biological properties including biocompatibility, cell differentiation, or reduce presumable toxic side effects. Thus, apart from this hybridization approach, the current trend that has been followed by researchers since the beginning of the millennium approximately is the replacement of these materials with polymers obtained from natural sources, as depicted in Figure 2, mainly owing to their significantly better biological properties, though their application in the biomedical field still involves several challenges, as will be discussed in the next subsection.

### 2.2. Natural Polymers

Biopolymers are usually derived from plants and animals [152,153]. These polymers have multiple functions within the body, e.g., the creation of tissues and providing molecules such as signals for the human endocrine system, among others [152,154]. Therefore, natural sources represent an ideal perspective for hydrogel fabrication, due to their well-known biological properties, although these materials are often limited due to the difficulty of their synthesis and process, as well as their poor stability and mechanical properties, which still remain the most challenging features of this materials [1,155,156]. The most common examples of biopolymers include polypeptides and polysaccharides (PSAs), as well as nucleic acids, which constitute DNA and RNA [152,157].

In addition, there is a current growing trend towards the use of bacteria-derived polymers to compete with conventional polymeric materials used for biomedical applications, due to their interesting biodegradability and biocompatibility. The production of these bacteria-derived polymers, which is based on bacterial fermentation by wild type or genetic engineered strains, has become a feasible and promising alternative due to its low cost. Microbial biopolymers include polysaccharides (hyaluronic acid, dextran and bacterial cellulose), polyamides (poly-γ-glutamic acid (PGA), poly-l-Lysine (PL)), polyesters (poly-3-hydroxybutyrate (PHB), PLA, PCL) and polyphosphates. These biodegradable, nontoxic, non-immunogenic and biocompatible characteristics of microbial polymers are the main reasons for them being considered as ideal candidates for biomedical applications, along with their cost-competitive processing, as was previously mentioned [158].

#### 2.2.1. Protein-Based Hydrogels

The advantages of hydrogels based on pure proteins are the simplicity of the synthesis process and the attainment of relatively uniform network structures, along with excellent biocompatibility [159]. However, there are some disadvantages that must be taken into account, for example, the difficulty of protein extraction and the high cost of these processes [160,161]. There are still lots of challenges that must be overcome for these types of hydrogels in order to achieve successful biomedical performances, such as understanding the structure and how it can change during the hydrogel synthesis process or once implanted within the body, complex high-scale production of protein-based hydrogels, solvents and cross-linking agents commonly used that can affect cell viability and cause cytotoxicity, designing of coiled coils as smart hydrogels or the limitation of adequate properties for specifical applications, such as the lack of electrical properties for heart tissue regeneration after myocardial infarction, which remains tremendously challenging [162]. Therefore, the most widely studied protein-based hydrogels are discussed.

Collagen (COL). COL represents 30% of protein content in vertebrate organisms [163]. It is the most abundant protein of the ECM, whose triple-helix structure provides great tensile strength [66,164]. There are different types of COL (type I, II, III, IV and V, among others) present depending on their function in the body [165]. Thus, due to its abundance in nature, availability (porcine, bovine and marine), biodegradability and biocompatibility and excellent cell viability values (>80%), COL is an optimal candidate for hydrogel manufacture for wound healing [66,71], TE [67,72], drug delivery [68] and ophthalmic applications [73]. Nevertheless, even though ex vivo assays with COL-based hydrogels have been carried out with human skin models [71], more in vivo experiments are necessary to characterize COL-based hydrogels for other applications, for example, bone tissue engineering [72]. Another advantage of COL is the possibility and capability to form gels by thermal condensation. However, their limited stability and mechanical properties hinder their performance as hydrogels, as they tend to degrade when cultured with cells [67]. This drawback can be overcome by cross-linking or blending processes. Grønlien et al. (2022) have reported that a COL-based hydrogel photo-cross-linked with lumichrome, a compound resulting from the photodegradation of riboflavin (vitamin B2) when irradiated in acidic and neutral solutions, which is more photostable than riboflavin, exhibited higher elasticity and thermal stability, along with an improved water storage capacity [69]. In addition, Mahou et al. (2018) developed hydrogels based on a COL−alginate blend. Comparing these to pure COL gels, COL−alginate gels were stiffer, which delayed their collagenase-caused degradation [70].

Gelatin (GEL). GEL is a derivative (a partially hydrolyzed form) of COL. The properties of GEL strongly depend on the processing of COL, its molecular weight and its isoelectric point. Obtaining GEL from COL requires breaking the bonds that stabilize the structure from a pre-treatment by means of acids, bases or enzymes. In this sense, type A GEL (isoelectric point 8–9) is obtained after an acid treatment of COL, and type B GEL (isoelectric point 4–5) is obtained with an alkaline treatment [74,152]. The amines present in the lysine side chains help the GEL to adhere to the carboxyl groups of the tissue surface molecules [75]. GEL hydrogels have been demonstrated to be advantageous due to their self-healing capacity, and have been used in injections for therapeutic purposes [76,152]. GEL has been proposed as an interesting alternative in hydrogel applications, since it overcomes several disadvantages of its parent compound, i.e., COL. For example, bare COL hydrogels may induce adverse immunological responses, along with a lack of mechanical and thermal stability [152,166]. For these reasons, as well as its abundance and availability, GEL has been extensively investigated for biomedical applications, although several approaches have been proposed to enhance its properties, for example, hybridizing with other polymers [77,78,167,168], cross-linking strategies [169,170] and chemical modification [79,80,171]. As to biological performances, GEL-based hydrogels have resulted in excellent candidates for biomedical applications since they have remarkable biocompatibility (>90%) and durability once implanted in the body, not only maintaining their cell viability, but even increasing after 7 days, as reported by Liu et al. (2022). Moreover, on the basis of good biocompatibility, the hybrid hydrogel was proven to exert a sufficient ability to promote cartilage regeneration by in vitro three-dimensional (3D) culture of adipose-derived stromal cells (rASCs) and in vivo articular cartilage defect repair on 6 weeks-old SD rats [79].

Polydopamine (PDA). PDA is a biopolymer that results from the oxidation and polymerization of dopamine, an analog compound of L-3,4-dihydroxyphenylalanine (DOPA), which is obtained from secreted marine mussel adhesive proteins by post-translational modification of tyrosine [83,84]. Moreover, another advantage of PDA is that it can be produced simply and cheaply without toxic solvents [85]. PDA provides key advantages for biomedical applications, since it is hydrophilic and capable of functionalizing different substrates [83]. Moreover, it has low cytotoxicity, excellent biocompatibility (>80%) and enhances cell adhesion and proliferation [172]. PDA-based hydrogels have been tested in vitro (with human renal epithelial cells, 293T) and in vivo with female mice for sterilization and wound healing applications, as reported by Gan et al. (2022), exhibiting excellent wound healing ability with minimal toxicity, thus indicating its great potential for use in wound dressings. [86]. Plus, ex vivo experiments have been carried out with human skin models for wound healing applications by O’Connor et al. (2020) with excellent results [87]. However, PDA-based hydrogels still need thorough research to improve their properties [88,89].

Elastin (EL). Elastin is an essential functional component of the dermal ECM and has demonstrated benefits in skin wound repair [90,173]. EL has superior mechanical resistance and structural stability in vivo [91]. The main function of elastin is to endow rubber-like elasticity to most of the body tissues. It has also been characterized as a highly insoluble polymer with remarkable cellular attachment, which enables cell growth and differentiation. Due to this highly cross-linked structure of elastin, tissues can undergo high deformation [91,174,175]. For these reasons, EL-based hydrogels have been posed as a prime biomaterial, with better mechanical properties than most biopolymeric hydrogels and good biocompatibility (>80%), although further research is required to optimize elastin-based hydrogels’ in vivo performance [92,94,95]. Unal et al. (2021) developed a hybrid GelMA/elastin-based composite hydrogel that showed excellent biodegradation ability in vivo when implanted subcutaneously in a murine animal model, as well as remarkable biocompatibility and mechanical properties with potential for vascular applications, specifically for endovascular anastomosis [94].

Thus, some of the most well-known protein materials such as COL, GEL and elastin have been described and, comparing these three materials, it can be concluded that COL is the most limited protein for hydrogel fabrication due to the strong influence of gelation conditions and poor physicomechanical features, especially compared to cross-linked COL-based hydrogels and GEL, because of GEL being the partially denatured structure of COL, which leads to an increase of the random coil content, favoring the structure for gelation. Nevertheless, GEL presents a poor thermomechanical demeanor, so structure modification and functionalization are necessary to obtain hydrogels with considerable properties for biomedical applications. On the other hand, elastin can be a very efficient biomaterial, due to its unique structure and stability. However, elastin still needs further research to improve it properties once introduced in the body [162,176].

There are lots of biopolymers that have not been mentioned, such as serum albumin [177], keratin [178], resilin [179] or silk [180], that have been thoroughly researched for hydrogel production for biomedical applications. This is mainly due to the fact that these materials share the same main drawbacks, which are basically the difficulty of large-scale production of these hydrogels and their poor mechanical properties [161,162]. Therefore, the current trends in protein-based hydrogels in order to overcome the challenges previously mentioned are to achieve stronger and more stable hydrogels by means of modifying these protein structures through cross-linking and functionalization strategies, decreasing the side effect of the toxic solvents and cross-linking agents, or to implement alternative ones that do not harm biological properties of the resulting hydrogels, and research new alternative proteins such as PDA from marine mussel to obtain novel protein-based hydrogels with different and promising properties for biomedical applications.

#### 2.2.2. Polysaccharide-Based (PSA) Hydrogels

PSAs are made up of a wide variety of different monosaccharide units that are covalently bonded via glycosidic bonds [181]. PSAs are important in the development of various plants, microorganisms and animals as framework constituents and energy suppliers. In addition, glycans also play a key role in some cell functions, such as adhesion and other cellular interactions, differentiation and signaling [182,183]. PSAs have proven to be excellent candidates for hydrogel manufacture due to their high stability and low cytotoxicity; they are usually more stable than proteins, since they are resistant at high temperature [152]. Moreover, the modification and functionalization of PSAs through different approaches is possible due to their structural diversity, which allows regulating the properties of PSA-based hydrogels, expanding their scope and making them even more interesting [182].

Still, there are several challenges that need to be overcome regarding polysaccharide-based hydrogels, such as directing hydrogel synthesis to greener methods employing low-energy consumption procedures and less hazardous solvents. Moreover, producing intelligent polysaccharide-based hydrogels for drug management, such as anti-cancer drugs, is still challenging. Plus, controlled biodegradation of polysaccharide-based hydrogels should undergo more in vivo investigation. As mentioned before, modifying polysaccharide-based hydrogels usually results in unique characteristics, such as higher elasticity and stretchability, and this results in the possibility of developing novel promising applications, such as smart hydrogels, flexible electronic devices, magnetic-responsive hydrogels and solid-state hydrogels [184,185]. Herein, some of the most used polysaccharides used for hydrogel engineering will be described.

Chitosan (CTS). CTS is obtained from partial deacetylation by strong, high-temperature alkali treatment of chitin, which is one of the most abundant natural amino polysaccharides in the world [97]. It is composed of repetitive and randomly distributed units of N-acetyl-D-glucosamine and D-glucosamine connected by β-(1,4) linkages [186]. CTS is also one of the most versatile biopolymers due to the ease and prospect of carrying out an extensive variety of modifications on its structure and, consequently, on its properties. Most of the CTS properties, such as its degradation rate and its physicochemical and biological properties, strongly depend on its molecular weight and its degree of deacetylation. Moreover, CTS-based hydrogels strongly depend on the processing conditions (pH, gelation temperature, concentration) [98]. Thus, CTS has been thoroughly studied for hydrogel manufacture, due to its structure, versatility and properties, such as good biocompatibility (>80%) [104], biodegradability, low toxicity and mucoadhesiveness, in addition to its anti-inflammatory, antibacterial, antifungal and wound-healing abilities [187]. Moreover, in vivo experiment results confirm the ability of CTS-based hydrogels to accelerate tissue regeneration to reduce inflammation and facilitate wound healing [102,103]. Current trends in CTS-based hydrogels for biomedical applications focus on the improvement of CTS performance and properties, from cross-linking strategies [99,100,101,188] to blending [189,190,191] and functionalization of CTS structure [192,193].

Hyaluronic acid (HA). This is a very well-known versatile biopolymer in the biomedical field, due to its sterling biodegradability, bioactivity, biocompatibility, non-thrombogenicity and non-immunogenicity. HA is a glycosaminoglycan present in the ECM of most tissues, most predominantly present in cartilage and ocular tissues [105,106]. It has a linear anionic polysaccharide, which consists of the repetition of N-acetyl-β-D-glucosamine and β-D-glucuronic acid units [105]. In addition, as HA structural and physicochemical properties strongly depend on its molecular weight (Mw), HA-based hydrogels’ properties can be easily modified to meet the specific application requirements. Some properties of HA can be modified by increasing its molecular weight or its concentration in aqueous solutions, which increases its pseudo-gelling and viscosity, due to chain entanglement and the formation of extensive hydrogen bonds (intra- and inter-molecularly) [106]. Nevertheless, HA exhibits an extremely rapid degradation rate, along with poor mechanical properties, hindering its potential use for some biomedical applications, such as TE. To overcome these drawbacks, numerous strategies have been proposed, for example, functionalization or cross-linking, with the aim of enhancing HA-based hydrogels’ stability and physicochemical properties [107]. Regarding biological properties, Long et al. (2022) performed in vitro biocompatibility studies with mouse fibroblast L929 cells, showing excellent biocompatibility (>90% cell viability for the tested hydrogels) and in vivo experiments with SD rats on an injectable multifunctional hydrogel based on dopamine-grafted hyaluronic acid and phenylboric acid-grafted methylcellulose. The obtained results revealed the great potential of the prepared system to significantly accelerate chronic wound repair [109].

Alginate (ALG). This is a commonly used biopolymer, based on a polysaccharide obtained from brown algae [111]. Its chemical structure is mainly composed of two different units, namely α-L-guluronic acid and β-D-mannuronic acid, connected by 1,4-glycosidic bonds [112]. Alginate is an abundant and available biopolymer that displays several interesting features, as it has remarkable biocompatibility, good porosity, great water retention capacity and tunable viscosity, which makes it a material particularly well-suited to biomedical approaches [111,113]. Alginates can form ionic gels due to ion exchange between the monovalent ion in the alginate solution and polyvalent cations [112]. However, as it has been proved that ionically cross-linked hydrogels degrade via ion exchange processes, involving ion loss, alginate may not be the most suitable biopolymer, as these processes usually cause uncontrolled dissolution of the polymeric network [194]. Alginates are commonly combined with other polymers to implement a synergistic effect in order to enhance their mechanical and biological performance as single polymers [195]. One example is the ALG-fibrinogen-based composite hydrogels developed by Soleimanpour et al. (2022) for skin wound healing. Results revealed that the synthesized system had excellent properties for wound healing applications due to its adequate mechanical features and biocompatibility. In addition, in vivo experiments on Winstar rat models showed that the fabricated biocomposite hydrogel could present an appropriate antibacterial effect and efficiency for chronic wound treatment [114].

Cellulose (CEL). Cellulose is obtained by D-type glucose polymerization. This monosaccharide comes from plants and bacteria. Cellulose-based hydrogels are a promising adsorbent biomaterial and present several advantages compared to other conventional synthetic adsorbents, due to their low cost and high abundance, considerable biocompatibility, biodegradability, nontoxicity, good thermal/chemical stability and excellent adsorption capacity. However, the main inconvenience of cellulose is that it cannot be used in its natural form like other biopolymers due to the abundance of hydroxyl groups; however, thanks to those groups, as well as other hydrophilic functional groups, namely carboxyl and aldehyde groups, it can be functionalized through several chemical reactions to form cellulose-based hydrogels [118,119,120]. Hu et al. (2022) developed an aminocelullose-dialdehyde xylan composite hydrogel with silver (Ag), synthesized via green method by Schiff-base reaction cross-linking, followed by an immersion step in silver nitrate solution, which exhibited excellent antibacterial properties against *E. coli* and promising wound healing performance [121]. On the other hand, Huang et al. (2022) developed a photo-cross-linked hydrogel from HA methacrylate and CMC methacrylate. The HA/CMC hydrogel had good biodegradability, biocompatibility (>80% cell viability for 3T3 fibroblasts in vitro cultured for 24 h) and mechanical properties. In vivo experiments demonstrated that these hydrogels can act properly as dural substitutes to repair dural defects in rabbits [126].

To sum up, polysaccharides are very good candidates for hydrogel production as they own several interesting features such as availability, abundance and low cost obtention, as well as excellent versatility and biological properties. However, polysaccharide-based hydrogels still present some improvable characteristics, such as the lack of antimicrobial activity and insufficient mechanical properties for hard-tissue engineering, among other applications [196]. Therefore, further research should be carried out in order to implement green synthesis procedures with nontoxic solvents not to harm the biological properties of the resulting hydrogels and exploring new functionalization, combination and cross-linking approaches seem mandatory to improve their properties and make them more suitable for biomedical applications including drug delivery, smart hydrogels, injectable hydrogels, tissue engineering and wound healing.

After considering all the characteristics and possibilities of the indicated synthetic and natural polymers, it is remarkable that there has been a significant advance in hydrogel engineering in the past two decades [197]. However, new challenges and applications have arisen and modern hydrogel investigation needs to search for possible solutions to adapt all the progress achieved to the new challenges [198]. Among all these possibilities, the hybridization of both natural and synthetic polymers to form hydrogels is the most common procedure, as it combines the advantages of each polymer type, offsetting drawbacks of single polymer-based hydrogels and providing suitable biological activity as well as better mechanical and biological properties [1].

## 3. Synthesis of Hydrogels

**Figure 3:**
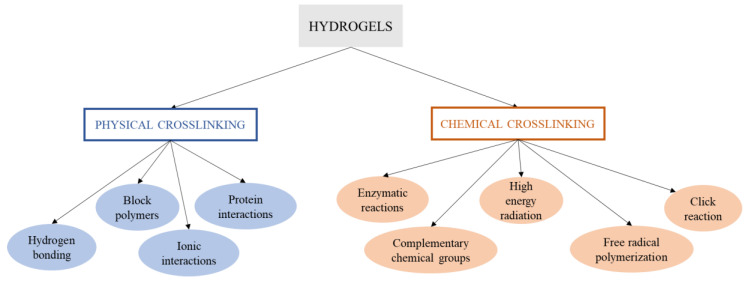
Schematic overview of the different physical and chemical cross-linking methods of synthesis of hydrogels.

As we commented in the Introduction, there are different methods to obtain hydrogels. A hydrogel is generally obtained by synthesis through the hydrolysis and condensation of the chosen precursors, causing the creation of a solid nanostructured network [199]. In this sense, for the synthesis of the hydrogel, most of the studies focus primarily on physical and chemical cross-linking methods (Figure 3), and the current challenges by means of synthesis procedures are oriented to obtain structures with higher cross-linking density for specific applications such as limbal stem cells [200] or hard-tissue engineering [201] and the use of green alternative procedures, solvents and cross-linking agents to assess efficient hydrogel synthesis without impairing biological properties such as biocompatibility and cytotoxicity [202,203,204].

### 3.1. Physical Cross-Linked Hydrogels

**Figure 4:**
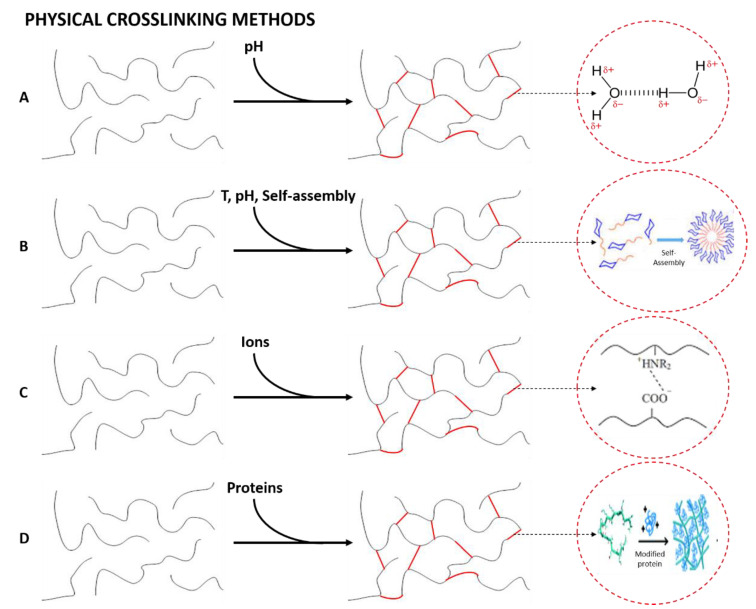
Formation of physically cross-linked hydrogels by (**A**) hydrogen bonding, (**B**) amphiphilic grafts and block polymers, (**C**) ionic interactions and (**D**) protein interactions.

When the liquid phase changes to a gel due to an environmental change (pH, temperature, mixing of two components or ionic concentration) the hydrogels formed are known as physical hydrogels [205]. Their main interest lies in the absence of cross-linking agents in the synthesis. Figure 4 shows schematically the formation of physically cross-linked networks, specifying the interaction that constitutes the mentioned structures for each of the four types of cross-links that generate physical hydrogels, as depicted in Figure 3 [206].

Hydrogen bonding. This cross-linking method is based on the formation of hydrogen bonds between the polymeric chains to form a nanostructured network [207]. However, Jing et al. (2022) showed that this type of bond has a strong dependence on the pH of the gel. In their study, they obtained pH-responsive alginate/chitosan hydrogels whose properties may be tuned depending on the pH of the solution [208].

Amphiphilic grafts and block polymers. This group is formed by molecules with the ability to self-assemble in aqueous solutions to form hydrogels and polymeric micelles, in which the hydrophobic part of the polymer is concentrated. Interestingly, block polymer-based hydrogels can also be formed via crystallization, as is shown by Castillo and Müller (2009) in their study about the use of crystallization to produce a block copolymer material with good hydrophilicity and suitable mechanical and physical properties to be used as potential biomaterials [209].

Ionic interactions. Hydrogel formation is favored by the presence of ions to form the internal network [210]. This method is normally carried out at room temperature and physiological pH. The resulting hydrogels are nontoxic, do not cause skin irritation, are easily extensible and have adequate adhesion strength to be applied as a polymeric film on the skin [211].

Protein interactions. This cross-linking method is based on the use of genetically modified proteins or through antigen−antibody interactions. The former is produced by modifying the peptide sequence, enabling the control of the hydrogels’ physicochemical properties. On the other hand, the addition of a free antigen as a cross-linking agent generates a slight swelling of the hydrogel due to the replacement of the antigen bound to the polymer, generating the release of antibodies together with a decrease in the cross-linking density [212].

### 3.2. Chemical Cross-Linked Hydrogels

**Figure 5:**
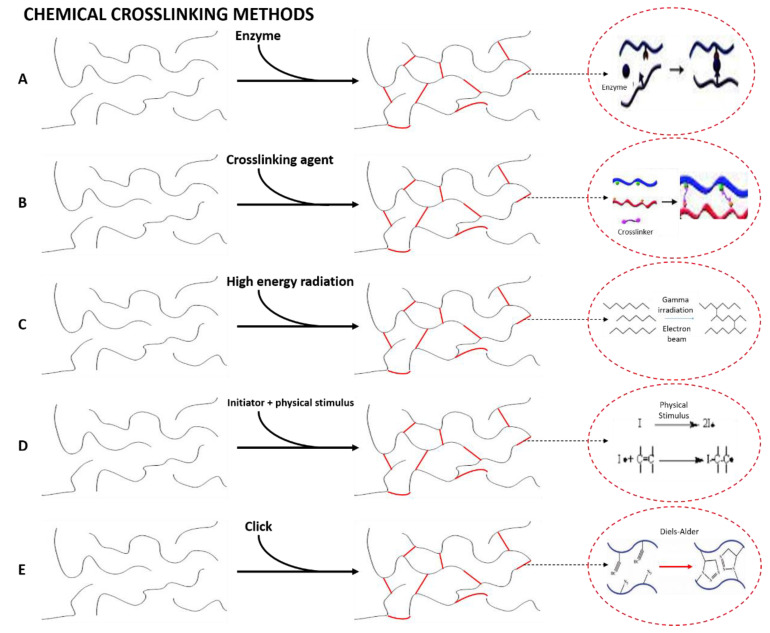
Formation of chemically cross-linked hydrogels by (**A**) enzymatic reactions, (**B**) chemical reaction promoted by a cross-linking agent, (**C**) high-energy radiation, (**D**) free-radical polymerization and (**E**) click reactions.

Chemical cross-linking is an irreversible process, where covalent bonds can be induced by the methods described below [15]. This type of hydrogel is of special interest thanks to their good mechanical resistance after cross-linking. For these hydrogels, there are mainly five ways to promote chemical cross-linking (Figure 3), whose specific interactions and conditions for the formation of the cross-linked structures are represented in Figure 5 [206].

Enzymatic reactions. Enzymatic cross-linking requires the use of enzymes as reactants in order to reduce the potential toxicity of the chemical reagents traditionally used [213]. For this reason, there is a controversy, since some authors have considered enzymatic cross-linking as a third cross-linking method, whereas other researchers include it as a specific type of chemical cross-linking. This type of cross-linking is generally carried out with biopolymers. Therefore, the formation of hydrogels can be favored by the presence of enzymes that act as additives to generate new bonds between the polymer chains [214]. In this line, transglutaminase is the most used enzyme to carry out this type of cross-linking [215].

Reaction of complementary chemical groups. These hydrogels are formed by the use of agents that generate secondary reactions to promote cross-linking of the hydrogel. This group includes aldehydes and those additives that promote condensation reactions [216].

High energy radiation. This cross-linking method is promoted by the use of gamma radiation or an electron beam. Recent studies have combined this cross-linking method with enzymes to produce a chemical gelling of hydrogels obtained with macromolecules [217].

Free-radical polymerization. The manufacture of hydrogels following this route requires the use of synthetic, semi-synthetic or natural hydrophilic polymers [218]. It is also necessary to use enzymes as catalysts to favor the reaction [219] or, most commonly, free radical initiators, which are compounds that can generate free radicals by different stimuli. Temperature, UV irradiation, oxidation, microwave irradiation and gamma radiation have been used to induce radical generation [220].

Click reactions. This term describes a type of rapid, spontaneous, versatile and extremely selective reactions that do not lead to the formation of secondary products and result in high yields of heteroatom-linked molecular systems with high efficiency in a wide variety of mild reaction conditions [221]. The “click chemistry” approach allows a wide variety of synthetic strategies to accomplish the cross-linking and chemical functionalization of hydrogels with tailored properties. Several well-known reactions, which can be classified into three groups, comply with the “click chemistry” approach for hydrogel manufacture. These three groups include: (1) copper-free click reactions, such as Diels–Alder (DA), strain-promoted azide-alkyne cycloaddition (SPAAC), oxime-forming reactions and radical mediated thiol-ene; (2) copper-catalyzed azide-alkyne cycloaddition (Cu-AAC), and (3) pseudo click reactions, which include aldehyde-hydrazide reactions (Schiff-base reactions) and thiol-Michael addition [176].

In this section, different synthesis procedures have been discussed for hydrogel manufacturing, leading to mainly two types of cross-linked structures, namely physical and chemical cross-linking. The main difference between these two cross-linking approaches is the formation or not of covalent bonds, respectively, in the resulting structure [222]. Thus, physically cross-linked hydrogels have some advantages, such as remarkable versatility and the absence of chemical compounds to attain the desired cross-linked structure, which commonly harms fundamental properties such as biocompatibility or causes toxicity problems once implanted within the body [205]. Nevertheless, as described previously, the main drawback of these methods is the reversible demeanor that they present and the strong dependence on environmental parameters such as pH or temperature, which leads to unstable properties. This is the main challenge for the physically cross-linked hydrogel, though they still represent a very interesting strategy to reinforce hydrogels’ structure [21].

On the other hand, chemical cross-linking methods stand out for the better stability and mechanical properties of the synthesized hydrogels, rather than physically cross-linked ones, due to the formation of covalent bonds. However, most of the compounds used to induce secondary reactions or to form free radicals, as well as common reagents for some click reactions are presumably toxic and negatively affect biological properties and induce cytotoxicity issues [223]. Therefore, the main challenge of these procedures would be to move towards the implementation of the abovementioned green alternative compounds to induce complementary group reactions or free radical initiators that do not harm the biological properties of the resulting systems [224,225]. In addition, further research to obtain specific enzymes would be another interesting approach to attain chemically cross-linked structures avoiding the use of toxic compounds [226,227].

The combination of cross-linking procedures and materials is apparently the best solution to obtain adequate hydrogel systems for biomedical applications [228]. Not only combining polymers, but also including other materials and further cross-linking stages could be beneficial in order to obtain suitable hydrogels for more specific and demanding biomedical applications. This statement will be discussed in the following section.

## 4. Hybrid Hydrogel Composites

To obtain desirable performances for biomedical applications, one of the most investigated approaches is the development of multicomponent composite hydrogels to gain distinct properties and functionalities [229]. Composite hydrogels result from the combination of different types of inorganic, organic and polymeric materials to achieve the synergistic effect of those materials for specific applications [25]. A wide number of inorganic and organic compounds, such as metallic or metal oxide nanoparticles, carbon-based, ceramic and polymeric materials, have been combined within hydrogel networks to attain a composite with tailored functionalities [229].

Composite hydrogels that include metal nanoparticles (NPs) and metal oxides are one of the most studied trends for the development of biomaterials, since these materials have beneficial properties, such as their ability to respond to physical stimuli (electrical, magnetic and light) and good antimicrobial activity [230]. Metallic nanoparticles mainly include noble metals such as silver (Ag) [231], gold (Au) [232] and platinum (Pt) [233], whereas metal oxide nanoparticles include titania (TiO_2_) [234], iron oxide (Fe_3_O_4_, Fe_2_O_3_) [235], zirconia (ZrO_2_) [236], alumina (Al_2_O_3_) [237] and zinc oxide (ZnO) [238]. The incorporation of noble metal NPs has indeed added advantageous functionality to hydrogels for biomedical applications. However, there are still several challenges that this technology must overcome, such as concerns about the cytotoxic effect of the metal NPs, because the mechanism has yet not been completely understood and the interaction of NPs with cells remains controversial. In addition, NP–hydrogel composites also present a critical challenge as massive uncontrolled release of NPs from the scaffold could take place once implanted in the body, causing undesired toxicity or other adverse effects, not only directly on implanted cells, but also translocated to other organs [239].

On the other hand, metal oxide NP hydrogel composites have been developed for their ferromagnetic and semiconducting properties. These types of hydrogels own the capacity to stimulate under a magnetic field and thus respond, suggesting that further research could lead to devices exhibiting human-like actuation for drug delivery and microfluidic valve control, though this investigation path still demands further research [240].

Composite hydrogels with ceramic materials are usually based on the incorporation of ceramic nanoparticles and nanotubes within hydrogel networks, designed especially for bone TE. These fillers can improve mechanical and biological characteristics of hydrogels. The main challenge when designing these nanocomposite hydrogels is the need to integrate the ceramic nanoparticles within the structure in order to achieve a multi-components network, as two-components systems are not able to incorporate multiple functionalities. Besides, further research on understanding the interactions between polymeric chains and nanoparticles at different length scales is imperative [241]. Among inorganic ceramic compounds, hydroxyapatite (Ca_10_(PO_4_)_6_(OH)_2_, HAp) is the most studied as it constitutes the largest portion of inorganic components in human bones and is typically used as a bone substitute. HAp can improve the percentage of local Ca^2+^, which can increase the proliferation of osteoblasts and promote the growth and differentiation of mesenchymal stem cells [242,243,244]. Hofmann et al. (2022) processed a new composite 2-hydroxyethyl methacrylate (HEMA)/gelatin/poly(β-amino ester) (PBAE) 3D scaffold with incorporated nHAp. This combination resulted in superior mechanical and functional properties, due to synergetic effects that also enhanced the interactions with biological species [242]. Regarding the addition of ceramic nanotubes, there is a recent trend of incorporating naturally occurring hollow nanotubes as a reinforcing agent in polymer composites, significantly improving mechanical properties such as stiffness and flexibility while maintaining good cytocompatibility and cell adhesion [245,246]. Among them, halloysite nanotubes (HNTs) are the most commonly reported ceramic nanotubes for this purpose, along with carbon nanotubes (CNTs), but these ones will be commented on later. The main reason is that HNTs are composed of hydrous aluminosilicate with a native tubular structure that can be easily obtained from natural resources. Along with its biocompatibility with human body, HNTs has been proposed as an interesting, valuable and low-cost alternative for bone tissue engineering applications [245,247]. Huang et al. (2019) reported the successful incorporation of HNTs into a GelMA hydrogel by photopolymerization process and that HNTs addition increased the mechanical performance and the capability to support cell adhesion and proliferation of human dental pulp stem cells [246]. In addition, another advantage of HNTs is the possibility of functionalization to further improve their performance as biomaterials, as reported by Naumenko et al. (2021), who modified HNTs with forskolin, a unique structurally complex labdane-type triterpenoid obtained from the plant Coleus forskohlii, belonging to the group of so-called small molecules, to obtain a new osteoconductive smart polymeric scaffold with improved osteodifferentiation [248].

Another type of hydrogel composite with ceramic material is the introduction of bioactive ceramic fillers or bioactive glasses into the structure of hydrogels. Dos Santos et al. (2021) studied the incorporation of HA into a CTS-biosilicate composite, increasing its water content capacity and cell cytocompatibility, with potential for dermal wound healing application [249].

Other materials that are of great interest in biomedical applications are those based on carbon (carbon-based nanomaterials, CBNs). Their interest comes from their unique physical and chemical properties, including structural, mechanical, electrical, optical and thermal properties. These special properties have allowed the development of new possible applications, as these carbon-based materials are combined with hydrogels to develop smart hydrogel systems, such as multifunctional photo-responsive hydrogels, though due to the novelty of these technologies, it is still necessary to carry out more studies to determine the toxicity of these systems to surrounding tissues, controlling biodegradation and research for a deep understanding of their photothermal mechanisms and properties [250]. Therefore, CBNs, such as graphene oxide (GO), CNTs and graphene quantum dots (GQDs) have been extensively studied in biomedical applications [251,252]. Guilbaud et al. (2022) studied hydrogels based on short homopeptides (Boc-α-diphenylalanine and Boc-α-dityrosine) containing light-responsive nanomaterials (CNTs and GO), which offered interesting results for the development of promising systems for controlled on-demand drug release applications [253]. Park et al. (2022) investigated the design and manufacture of three types of hybrid poly(NIPAAm-co-BBVIm) (pNIBBIm) and CTS hydrogels with incorporated CNTs for smart drug delivery systems, with remarkable thermal and electrical responsive properties that enabled the development of an excellent controllable and switchable drug delivery platform for biomedical approaches [254]. Kim et al. (2021) synthesized chemically flexible and mechanically tough PAA hydrogels using GQDs as a photo-initiator and confirmed that the unique photochemical properties of GQDs triggered the efficient cross-linking process of acrylamide monomers, enhancing their mechanical resistance and swelling behavior [255]. Finally, Mamidi et al. synthesized carbon nano-onions to reinforce ultra-high molecular weight polyethylene (UHMWPE) [256] and gelatin hydrogels [257,258].

Among composite hydrogels based on polymers, the incorporation of interpenetrating secondary networks with the aim to improve the properties of polymeric and biopolymeric hydrogels has posed a revolution in polymer and hydrogel science and, thus, the main research line in this field. Interpenetrating polymer networks (IPN) are the combination of independent, yet interdigitating polymer networks at the molecular level [259]. According to the preparation parameters, IPN hydrogels can be classified as: (i) simultaneous IPN, when the precursors of both networks are mixed, resulting in the formation of the two networks at the same time, or (ii) sequential IPN, typically performed by swelling a single-network hydrogel into a solution containing the mixture of monomer, initiator and activator, with or without cross-linking agent. With respect to their structures, IPN hydrogels can be classified as: (i) IPNs or full-IPNs, which are polymer matrices composed of two cross-linked networks interconnected on a molecular scale, or (ii) semi-IPNs, which are matrices consisting of only one cross-linked network, in which the linear or branched polymer penetrates the network [260].

The main difference between IPN hydrogels and conventional composite hydrogels is that networks conforming an IPN system cannot be separated without cleaving crosslinks. IPN hydrogels may be preferred over polymer blends due to their improved mechanical strength, controlled swelling behavior and efficient drug loading capacity [259,261]. One of the most used biopolymers for IPN formation is CTS, as reported by Dragan et al. (2020) [262], although there have been a large number of research papers based on IPN hydrogels synthesized with different synthetic and natural polymers, such as COL [263,264,265], GEL [266,267,268], alginate [267,269,270], polyurethane [264,265,270], PVA [268,271], PEG [272,273] and poly (aspartic acid) [274], among other candidate polymers. In this sense, there is a wide range of possibilities by means of materials and synthesis procedures that can be used for IPNs formation, owing to their outstanding physicochemical properties. Nevertheless, more research has to be accomplished in order to understand properly the sorption and transport mechanisms and more in vivo studies should be carried out to assess IPN’s biological properties and implement them for biomedical application. In fact, IPN systems present remarkable properties for drug delivery systems [275].

In conclusion, the incorporation of different materials within the structure of hydrogels opened the path for new research lines for the development of different hydrogel applications due to the introduction of new properties such as thermal, electric and magnetic response capacities, as well as significantly improving some well-known properties such as antibacterial activity when metal NPs were incorporated, or mechanical properties in the case of metal oxide and ceramic NPs or when another polymeric network was introduced during IPN formation. These novel capacities of hydrogels will lead to future investigations of the extension of the range of applications, as well as improving their performance for other popular biomedical lines such as tissue engineering, wound healing and drug delivery. Nevertheless, in this section, several challenges have been discussed for each composite system, indicating that there is still much more investigation work to carry out in order to attain large-scale commercial application of these novel technologies.

## 5. Applications of Hydrogels and Future Perspectives

**Figure 6:**
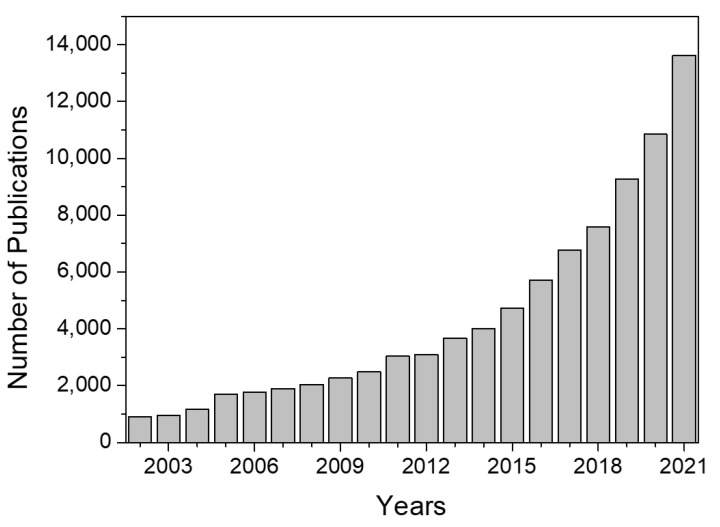
Evolution of the number of publications related to hydrogels. Data obtained from Scopus.

The research field of hydrogels is evolving and gaining recognition over the years. Figure 6 shows the evolution of the number of publications concerning hydrogels since 2002. As can be observed, the evolution follows an exponential trend, especially from 2018.

This growing evolution is based on the versatility of hydrogels, which lies in the fact that their properties and, therefore, their possible applications can be modified depending on the type of cross-linking (physical or chemical), nature of the polymer, cross-linking agent, combination with other materials, and size of the system (hydrogels, microgels, nanogels, etc.), among other characteristics. For instance, their structure (high content in water), degradation and release capacity make them suitable candidates as water reservoirs in agriculture or horticulture for those areas where there is a shortage of water for crop irrigation [276]. However, their main physical and biopharmaceutical characteristics such as swelling, mucoadhesiveness and the particularities of the release of the drugs housed inside them make them potential candidates in numerous biomedical applications, such as systems for wound dressing, the controlled release of drugs, TE, contact lenses and hygiene products [277,278].

Currently, there are many different approaches to the manufacture and design of hydrogel systems for diverse applications, in addition to those previously mentioned, whose main purpose is to enhance hydrogels’ properties for better performance. As was previously discussed, due to the poor mechanical properties of traditional hydrogels, as well as their low stability and inability to completely replicate important aspects of the cellular microenvironment, they cannot be easily applied in biomedicine. As was mentioned in the previous section, the incorporation of IPN has raised as an excellent solution to overcome these limitations [259]. Nevertheless, there are other composite approaches that can meet these requirements. For example, the incorporation of bioactive glasses to form composite hydrogels has been highly studied due to the bioactivity, biocompatibility and osteogenic effects that could be conferred to these materials [229].

Other perspectives focus on the research of several new material sources [158] or novel applications. In this sense, bioprinting has experienced great development in recent years [279]. In this field, hydrogels are widely used as bioinks to manufacture scaffolds for the regeneration of complex tissue structures [280]. In this way, the properties of hydrogels (such as their porosity and viscosity) make them the ideal material for this type of applications, since they allow housing cells and they can flow and give rise to the specific previously designed scaffold. Nevertheless, there are still several limitations that must be taken into account, especially for bioinks, which should own unique properties such as structural stability, cell growth promotion and a degradation rate consistent with tissue regeneration and they must be incorporated with cells. In addition, they should also be compatible with printers for rapid prototyping with high precision. Currently, bioinks have been limited in their ability to meet all of these needs, and a desirable bioink has yet to be identified. Moreover, other challenges of this field are the management of the sterile surgical field and time during the bioprinting process of the constructs as it may take longer to develop 3D constructs than cells’ survival time and it is difficult to maintain sterility during the operative process, as well as other limitations, including in vivo performance within the body and ethical issues that must be overcome [281,282].

The development of the so-called smart hydrogels as functional materials has been a revolution in this field of study, concerning responsive materials known as stimuli-responsive hydrogels. In response to external stimuli, these smart hydrogels can undergo structural and volume phase transitions, providing enormous potential for multidimensional technological applications. Generally, smart hydrogels consist of absorbent to superabsorbent materials that can react to any subtle environmental changes such as pH, temperature, electric field, ionic strength, chemical species and biological condition [25]. Even though smart hydrogels are a promising guideline to develop novel technologies, there is still much more work that must be carried out, especially for in vivo functional studies, which are only dedicated to animals. From a future perspective, there is another issue that these technologies must overcome, as contemporary hydrogels are mainly designed for a single purpose, but in practical biomedical applications, more work needs to be done [185,283].

In addition, hydrogels have proved to be a novel tool for developing the biomimetic lure-and-kill approach for pest management, as they can satisfy multiple needs simultaneously. Some attractive issues of this approach are the potential for applicability to several pest species, along with its cost-competitiveness. Moreover, it is already technically feasible, since all the technologies necessary to design and synthesize materials with these specific properties are already available on the market. Nevertheless, this novel application is still in its infancy and needs thorough research, as developing a new pest control approach requires a multidisciplinary process and strong interaction among different research areas. In fact, one of the possible limitations could be finding specific behavioral patterns in insects and quantifying their driver parameters, as well as designing the hydrogel with adequate properties for each case [284].

## 6. Conclusions

In this review article, authors have intended to contextualize the state-of-art of hydrogel science for biomedical applications. As a concluding remark, there has been an enormous development in hydrogel engineering in the past few years, as thorough research work has been done to discover new materials with interesting properties for hydrogel fabrication and combinations with other more well-known ones to improve hydrogels properties. New strategies have been developed by means of synthesis and cross-linking procedures and the incorporation of different materials such as metallic and non-metallic NPs or secondary interpenetrated polymeric networks to implement novel functionalities and substantially improve physicochemical and biological properties. Due to these new approaches, new applications have arisen. However, there are still too many challenges that hydrogels must overcome in all disciplines, as discussed throughout this review, which can be summarized in two main issues, regardless of the application that they would be designed for, namely the need for more in vivo investigation to know how the new approaches and devices interact with animals and, subsequently, in human bodies once implanted, as well as the need to develop novel efficient and environmentally friendly methods toward synthesizing hydrogels, avoiding the use of toxic solvents and cross-linking agents that would harm biocompatibility and other biological properties.

## Figures and Tables

**Table 1:** Summary of studies carried out with different synthetic and natural polymers.

Type of Polymer	Main Polymer	Experimental Stage	Cell Viability (%)	References
Synthetic Polymers	pHEMA	In vitro (human), in vivo (mice and porcine)	>80	[39,40,41,42,43,44,45]
	PVA	In vitro (human), in vivo (mice) Ex vivo (porcine)	>88	[46,47,48,49,50,51,52]
	PEG	In vitro (human), in vivo (mice) Ex vivo (porcine)	>80	[53,54,55,56,57,58]
	pNIPAAm	In vitro (human), ex vivo (porcine)	>90	[59,60,61]
	PNVCL	In vitro (human)	>85	[62,63,64,65]
Natural Polymers	Collagen (COL)	In vitro (human), in vivo (mice) Ex vivo (human)	>80	[66,67,68,69,70,71,72,73]
	Gelatin (GEL)	In vitro (human), in vivo (mice) Ex vivo (mice)	>90	[74,75,76,77,78,79,80,81,82]
	Polidopamine (PDA)	In vitro (human), in vivo (mice) Ex vivo (human)	>80	[83,84,85,86,87,88,89]
	Elastin (EL)	In vitro (human), in vivo (mice) Ex vivo (porcine)	>80	[90,91,92,93,94,95,96]
	Chitosan (CTS)	In vitro (human), in vivo (mice) Ex vivo (human)	>80	[97,98,99,100,101,102,103,104]
	Hyaluronic Acid (HA)	In vitro (human), in vivo (mice) Ex vivo (human)	>80	[104,105,106,107,108,109,110]
	Alginate (ALG)	In vitro (human), in vivo (mice) Ex vivo (porcine and canine)	>85	[111,112,113,114,115,116,117]
	Cellulose (CEL)	In vitro (human), in vivo (mice) Ex vivo (porcine)	>75	[118,119,120,121,122,123,124,125,126]

## Data Availability

Not applicable.

## References

[1] Radulescu D.M., Neacsu I.A., Grumezescu A.M., Andronescu E. (2022). New Insights of Scaffolds Based on Hydrogels in Tissue Engineering. Polymers.

[2] Martin I., Wendt D., Heberer M. (2004). The role of bioreactors in tissue engineering. Trends Biotechnol..

[3] Cunniffe G.M., O’Brien F.J. (2011). Collagen scaffolds for orthopedic regenerative medicine. Jom.

[4] Vacanti J.P., Langer R. (1999). Tissue engineering: The design and fabrication of living replacement devices for surgical reconstruction and transplantation. Lancet.

[5] Liu X., Wu K., Gao L., Wang L., Shi X. (2022). Biomaterial strategies for the application of reproductive tissue engineering. Bioact. Mater..

[6] Li J., Mooney D.J. (2016). Designing hydrogels for controlled drug delivery. Nat. Rev. Mater..

[7] Cai L., Dewi R.E., Heilshorn S.C. (2015). Injectable hydrogels with in situ double network formation enhance retention of transplanted stem cells. Adv. Funct. Mater..

[8] Aldana A.A., Abraham G.A. (2017). Current advances in electrospun gelatin-based scaffolds for tissue engineering applications. Int. J. Pharm..

[9] Eltom A., Zhong G., Muhammad A. (2019). Scaffold Techniques and Designs in Tissue Engineering Functions and Purposes: A Review. Adv. Mater. Sci. Eng..

[10] Khanna A., Zamani M., Huang N.F. (2021). Extracellular matrix-based biomaterials for cardiovascular tissue engineering. J. Cardiovasc. Dev. Dis..

[11] Ding X., Zhao H., Li Y., Lee A.L., Li Z., Fu M., Li C., Yang Y.Y., Yuan P. (2020). Synthetic peptide hydrogels as 3D scaffolds for tissue engineering. Adv. Drug Deliv. Rev..

[12] Arifin N., Sudin I., Hasrul N., Ngadiman A. (2022). A Comprehensive Review of Biopolymer Fabrication in Additive Manufacturing Processing for 3D-Tissue-Engineering Scaffolds. Polymers.

[13] Neves S.C., Moroni L., Barrias C.C., Granja P.L. (2020). Leveling Up Hydrogels: Hybrid Systems in Tissue Engineering. Trends Biotechnol..

[14] El Blidi O., El Omari N., Balahbib A., Ghchime R., El Menyiy N., Ibrahimi A., Kaddour K.B., Bouyahya A., Chokairi O., Barkiyou M. (2021). Extraction methods, characterization and biomedical applications of collagen: A review. Biointerface Res. Appl. Chem..

[15] Chaudhary S., Chakraborty E. (2022). Hydrogel based tissue engineering and its future applications in personalized disease modeling and regenerative therapy. Beni-Suef Univ. J. Basic Appl. Sci..

[16] Ahmed E.M. (2015). Hydrogel: Preparation, characterization, and applications: A review. J. Adv. Res..

[17] Caló E., Khutoryanskiy V.V. (2015). Biomedical applications of hydrogels: A review of patents and commercial products. Eur. Polym. J..

[18] Ahmad S., Ahmad M., Manzoor K., Purwar R., Ikram S. (2019). A review on latest innovations in natural gums based hydrogels: Preparations & applications. Int. J. Biol. Macromol..

[19] Volpi M., Paradiso A., Costantini M., Wojciech S. (2022). Hydrogel-Based Fiber Biofabrication Techniques for Skeletal Muscle Tissue Engineering. ACS Biomater. Sci. Eng..

[20] Hoffman A.S. (2012). Hydrogels for biomedical applications. Adv. Drug Deliv. Rev..

[21] Bustamante-torres M., Romero-fierro D., Arcentales-vera B., Palomino K. (2021). Hydrogels Classification According to the Physical or Chemical Interactions and as Stimuli-Sensitive Materials. Gels.

[22] Worthington P., Pochan D.J., Langhans S.A. (2015). Peptide hydrogels—Versatile matrices for 3D cell culture in cancer medicine. Front. Oncol..

[23] Yahia L.H. (2015). History and Applications of Hydrogels. J. Biomed. Sci..

[24] Xie R., Zheng W., Guan L., Ai Y., Liang Q. (2020). Engineering of Hydrogel Materials with Perfusable Microchannels for Building Vascularized Tissues. Small.

[25] Sikdar P., Uddin M.M., Dip T.M., Islam S., Hoque M.S., Dhar A.K., Wu S. (2021). Recent advances in the synthesis of smart hydrogels. Mater. Adv..

[26] Pita-López M.L., Fletes-Vargas G., Espinosa-Andrews H., Rodríguez-Rodríguez R. (2021). Physically cross-linked chitosan-based hydrogels for tissue engineering applications: A state-of-the-art review. Eur. Polym. J..

[27] Rodríguez-Rodríguez R., Espinosa-Andrews H., Velasquillo-Martínez C., García-Carvajal Z.Y. (2020). Composite hydrogels based on gelatin, chitosan and polyvinyl alcohol to biomedical applications: A review. Int. J. Polym. Mater. Polym. Biomater..

[28] Tang G., Tan Z., Zeng W., Wang X., Shi C., Liu Y., He H., Chen R., Ye X. (2020). Recent Advances of Chitosan-Based Injectable Hydrogels for Bone and Dental Tissue Regeneration. Front. Bioeng. Biotechnol..

[29] Sharma S., Tiwari S. (2020). A review on biomacromolecular hydrogel classification and its applications. Int. J. Biol. Macromol..

[30] Catoira M.C., Fusaro L., Di Francesco D., Ramella M., Boccafoschi F. (2019). Overview of natural hydrogels for regenerative medicine applications. J. Mater. Sci. Mater. Med..

[31] Oryan A., Kamali A., Moshiri A., Baharvand H., Daemi H. (2018). Chemical crosslinking of biopolymeric scaffolds: Current knowledge and future directions of crosslinked engineered bone scaffolds. Int. J. Biol. Macromol..

[32] Schmidt B.V.K.J. (2019). Hydrophilic polymers. Polymers..

[33] Chai Q., Jiao Y., Yu X. (2017). Hydrogels for biomedical applications: Their characteristics and the mechanisms behind them. Gels.

[34] Mehrotra D., Dwivedi R., Nandana D., Singh R.K. (2020). From injectable to 3D printed hydrogels in maxillofacial tissue engineering: A review. J. Oral Biol. Craniofacial Res..

[35] Yang Y., Xu L., Wang J., Meng Q., Zhong S., Gao Y., Cui X. (2022). Recent advances in polysaccharide-based self-healing hydrogels for biomedical applications. Carbohydr. Polym..

[36] Huang B., Li P., Chen M., Peng L., Luo X., Tian G., Wang H., Wu L., Tian Q., Li H. (2022). Hydrogel composite scaffolds achieve recruitment and chondrogenesis in cartilage tissue engineering applications. J. Nanobiotechnol..

[37] Seo H.S., Wang C.P.J., Park W., Park C.G. (2022). Short Review on Advances in Hydrogel-Based Drug Delivery Strategies for Cancer Immunotherapy. Tissue Eng. Regen. Med..

[38] Simpson L.W., Good T.A., Leach J.B. (2020). Protein folding and assembly in confined environments: Implications for protein aggregation in hydrogels and tissues. Biotechnol. Adv..

[39] Zare M., Bigham A., Zare M., Luo H., Rezvani Ghomi E., Ramakrishna S. (2021). Phema: An overview for biomedical applications. Int. J. Mol. Sci..

[40] Saptaji K., Iza N.R., Widianingrum S., Mulia V.K., Setiawan I. (2021). Poly(2-hydroxyethyl methacrylate) hydrogels for contact lens applications–a review. Makara J. Sci..

[41] Chen Y., Zhang S., Cui Q., Ni J., Wang X., Cheng X., Alem H., Tebon P., Xu C., Guo C. (2020). Microengineered poly(HEMA) hydrogels for wearable contact lens biosensing. Lab Chip.

[42] Bhat A., Amanor-Boadu J.M., Guiseppi-Elie A. (2020). Toward Impedimetric Measurement of Acidosis with a pH-Responsive Hydrogel Sensor. ACS Sens..

[43] Bhat A., Smith B., Dinu C.Z., Guiseppi-Elie A. (2019). Molecular engineering of poly(HEMA-co-PEGMA)-based hydrogels: Role of minor AEMA and DMAEMA inclusion. Mater. Sci. Eng. C.

[44] Wang Y., Ouyang H., Xie Y., Jiang Y., Zhao L., Peng W., Wu J., Bao J., Liu Y., Wu J. (2022). Mechanically robust, biocompatible, and durable PHEMA-based hydrogels enabled by the synergic effect of strong intermolecular interaction and suppressed phase separation. Polymer.

[45] Kim Y.W., Kim J.E., Jung Y., Sun J.Y. (2019). Non-swellable, cytocompatible pHEMA-alginate hydrogels with high stiffness and toughness. Mater. Sci. Eng. C.

[46] Pan H., Fan D., Duan Z., Zhu C., Fu R., Li X. (2019). Non-stick hemostasis hydrogels as dressings with bacterial barrier activity for cutaneous wound healing. Mater. Sci. Eng. C.

[47] Luo C., Huang M., Sun X., Wei N., Shi H., Li H., Lin M., Sun J. (2022). Super-Strong, Nonswellable, and Biocompatible Hydrogels Inspired by Human Tendons. ACS Appl. Mater. Interfaces.

[48] Kodavaty J. (2022). Poly (vinyl alcohol) and hyaluronic acid hydrogels as potential biomaterial systems—A comprehensive review. J. Drug Deliv. Sci. Technol..

[49] Oh G.W., Choi I.W., Park W.S., Oh C.H., Heo S.J., Kang D.H., Jung W.K. (2022). Preparation and properties of physically cross-linked PVA/pectin hydrogels blended at different ratios for wound dressings. J. Appl. Polym. Sci..

[50] Kim M.S., Oh G.W., Jang Y.M., Ko S.C., Park W.S., Choi I.W., Kim Y.M., Jung W.K. (2020). Antimicrobial hydrogels based on PVA and diphlorethohydroxycarmalol (DPHC) derived from brown alga Ishige okamurae: An In Vitro and In Vivo study for wound dressing application. Mater. Sci. Eng. C.

[51] Tummala G.K., Joffre T., Lopes V.R., Liszka A., Buznyk O., Ferraz N., Persson C., Griffith M., Mihranyan A. (2016). Hyperelastic Nanocellulose-Reinforced Hydrogel of High Water Content for Ophthalmic Applications. ACS Biomater. Sci. Eng..

[52] Tummala G.K., Lopes V.R., Mihranyan A., Ferraz N. (2019). Biocompatibility of nanocellulose-reinforced PVA hydrogel with human corneal epithelial cells for ophthalmic applications. J. Funct. Biomater..

[53] Sabel-Grau T., Tyushina A., Babalik C., Lensen M.C. (2022). UV-VIS Curable PEG Hydrogels for Biomedical Applications with Multifunctionality. Gels.

[54] Chen T., Wang Y., Xie J., Qu X., Liu C. (2022). Lysozyme Amyloid Fibril-Integrated PEG Injectable Hydrogel Adhesive with Improved Antiswelling and Antibacterial Capabilities. Biomacromolecules.

[55] Stocke N.A., Zhang X., Hilt J.Z., DeRouchey J.E. (2017). Transport in PEG-Based Hydrogels: Role of Water Content at Synthesis and Crosslinker Molecular Weight. Macromol. Chem. Phys..

[56] Dethe M.R., Prabakaran A., Ahmed H., Agrawal M., Roy U., Alexander A. (2022). PCL-PEG copolymer based injectable thermosensitive hydrogels. J. Control. Release.

[57] Gökçe Kocabay Ö., İsmail O. (2021). Biodegradable Thermosensitive Injectable Poly(ε-caprolactone)–Poly(ethylene glycol)–Poly(ε-caprolactone) Based Hydrogels for Biomedical Applications. Polym. Sci.-Ser. A.

[58] Liu S., Jiang T., Guo R., Li C., Lu C., Yang G., Nie J., Wang F., Yang X., Chen Z. (2021). Injectable and Degradable PEG Hydrogel with Antibacterial Performance for Promoting Wound Healing. ACS Appl. Bio Mater..

[59] Yu Y., Cheng Y., Tong J., Zhang L., Wei Y., Tian M. (2021). Recent advances in thermo-sensitive hydrogels for drug delivery. J. Mater. Chem. B.

[60] Song X., Zhang Z., Zhu J., Wen Y., Zhao F., Lei L., Phan-Thien N., Khoo B.C., Li J. (2020). Thermoresponsive Hydrogel Induced by Dual Supramolecular Assemblies and Its Controlled Release Property for Enhanced Anticancer Drug Delivery. Biomacromolecules.

[61] Christiani T., Mys K., Dyer K., Kadlowec J., Iftode C., Vernengo A.J. (2021). Using embedded alginate microparticles to tune the properties of in situ forming poly(N-isopropylacrylamide)-graft-chondroitin sulfate bioadhesive hydrogels for replacement and repair of the nucleus pulposus of the intervertebral disc. JOR Spine.

[62] Gonzalez-urias A., Licea-claverie A., Sañudo-barajas J.A., Gonz M.A. (2022). NVCL-Based Hydrogels and Composites for Biomedical Applications: Progress in the Last Ten Years. Int. J. Mol. Sci..

[63] Ramos J., Imaz A., Forcada J. (2012). Temperature-sensitive nanogels: Poly(N-vinylcaprolactam) versus poly(N-isopropylacrylamide). Polym. Chem..

[64] Cortez-Lemus N.A., Licea-Claverie A. (2018). Preparation of a mini-library of thermo-responsive star (NVCL/NVP-VAc) polymers with tailored properties using a hexafunctional xanthate RAFT agent. Polymers.

[65] Durkut S., Elçin Y.M. (2020). Synthesis and Characterization of Thermosensitive Poly(N-Vinyl Caprolactam)-Grafted-Aminated Alginate Hydrogels. Macromol. Chem. Phys..

[66] Sharma S., Kumar V., Narang R.K., Markandeywar T.S. (2022). Collagen-based formulations for wound healing: A literature review. Life Sci..

[67] Gomez-Florit M., Pardo A., Domingues R.M.A., Graça A.L., Babo P.S., Reis R.L., Gomes M.E. (2020). Natural-Based Hydrogels for Tissue Engineering Applications. Molecules.

[68] Nguyen C.T., Vu M.Q., Phan T.T., Vu T.Q., Vo Q.A., Bach G.L., Thai H. (2020). Novel pH-Sensitive Hydrogel Beads Based on Carrageenan and Fish Scale Collagen for Allopurinol Drug Delivery. J. Polym. Environ..

[69] Gjestvang K., Elisabeth M., Beate S., Therese N., Hjorth H. (2022). Tuning of 2D cultured human fibroblast behavior using lumichrome photocrosslinked collagen hydrogels. Mater. Today Commun..

[70] Mahou R., Vlahos A.E., Shulman A., Sefton M.V. (2018). Interpenetrating Alginate-Collagen Polymer Network Microspheres for Modular Tissue Engineering. ACS Biomater. Sci. Eng..

[71] Hauck S., Zager P., Halfter N., Wandel E., Torregrossa M., Kakpenova A., Rother S., Ordieres M., Räthel S., Berg A. (2021). Collagen/hyaluronan based hydrogels releasing sulfated hyaluronan improve dermal wound healing in diabetic mice via reducing inflammatory macrophage activity. Bioact. Mater..

[72] Li Y., Liu Y., Li R., Bai H., Zhu Z., Zhu L., Zhu C., Che Z., Liu H., Wang J. (2021). Collagen-based biomaterials for bone tissue engineering. Mater. Des..

[73] Kong B., Sun L., Liu R., Chen Y., Shang Y., Tan H., Zhao Y., Sun L. (2022). Recombinant human collagen hydrogels with hierarchically ordered microstructures for corneal stroma regeneration. Chem. Eng. J..

[74] Salahuddin B., Wang S., Sangian D., Aziz S., Gu Q. (2021). Hybrid Gelatin Hydrogels in Nanomedicine Applications. ACS Appl. Bio Mater..

[75] Hazrati R., Davaran S., Omidi Y. (2022). Bioactive functional scaffolds for stem cells delivery in wound healing and skin regeneration. React. Funct. Polym..

[76] Sisso A.M., Boit M.O., DeForest C.A. (2020). Self-healing injectable gelatin hydrogels for localized therapeutic cell delivery. J. Biomed. Mater. Res.-Part A.

[77] Hwang J., Le Thi P., Lee S., Park E.H., Lee E., Kim E., Chang K., Park K.D. (2022). Injectable gelatin-poly(ethylene glycol) adhesive hydrogels with highly hemostatic and wound healing capabilities. J. Ind. Eng. Chem..

[78] Zulkiflee I., Fauzi M.B. (2021). Gelatin-polyvinyl alcohol film for tissue engineering: A concise review. Biomedicines.

[79] Liu F., Wang X., Li Y., Ren M., He P., Wang L., Xu J., Yang S., Ji P. (2022). Dendrimer-modified gelatin methacrylate hydrogels carrying adipose-derived stromal/stem cells promote cartilage regeneration. Stem Cell Res. Ther..

[80] Zhu S., Yu C., Liu N., Zhao M., Chen Z., Liu J., Li G., Huang H., Guo H., Sun T. (2022). Injectable conductive gelatin methacrylate / oxidized dextran hydrogel encapsulating umbilical cord mesenchymal stem cells for myocardial infarction treatment. Bioact. Mater..

[81] Hozumi T., Kageyama T., Ohta S., Fukuda J., Ito T. (2018). Injectable Hydrogel with Slow Degradability Composed of Gelatin and Hyaluronic Acid Cross-Linked by Schiff’s Base Formation. Biomacromolecules.

[82] Agten H., Van Hoven I., Ribeiro Viseu S., Van Hoorick J., Van Vlierberghe S., Luyten F., Bloemen V. (2022). In Vitro and In Vivo Evaluation of 3D Constructs Engineered with Human iPSC-Derived 2 Chondrocytes in Gelatin-Methacryloyl Hydrogel. Biotechnol. Bioeng..

[83] Vale A.C., Pereira P.R., Alves N.M. (2021). Polymeric biomaterials inspired by marine mussel adhesive proteins. React. Funct. Polym..

[84] Batul R., Tamanna T., Khaliq A., Yu A. (2017). Recent progress in the biomedical applications of polydopamine nanostructures. Biomater. Sci..

[85] Li H., Yin D., Li W., Tang Q., Zou L., Peng Q. (2021). Polydopamine-based nanomaterials and their potentials in advanced drug delivery and therapy. Colloids Surfaces B Biointerfaces.

[86] Gan Y., Lin C., Zhu H., Cheng X., Liu C., Shi J. (2022). An injectable self-healing CS/PDA-AgNPs hybrid hydrogel for mild and highly-efficient photothermal sterilization. New J. Chem..

[87] O’Connor N.A., Syed A., Wong M., Hicks J., Nunez G., Jitianu A., Siler Z., Peterson M. (2020). Polydopamine antioxidant hydrogels for wound healing applications. Gels.

[88] Zhang W., Huang Y., Wu H., Dou Y., Li Z., Zhang H. (2022). Polydopamine-heparin complex reinforced antithrombotic and antimicrobial activities of heparinized hydrogels for biomedical applications. Compos. Part A Appl. Sci. Manuf..

[89] Zhu S., Gu Z., Xiong S., An Y., Liu Y., Yin T., You J., Hu Y. (2016). Fabrication of a novel bio-inspired collagen-polydopamine hydrogel and insights into the formation mechanism for biomedical applications. RSC Adv..

[90] Sánchez-Cid P., Perez-Puyana V., Jiménez-Rosado M., Guerrero A., Romero A. (2021). Influence of elastin on the properties of hybrid PCL/elastin scaffolds for tissue engineering. J. Appl. Polym. Sci..

[91] Sharma A., Sharma P., Roy S. (2021). Elastin-inspired supramolecular hydrogels: A multifaceted extracellular matrix protein in biomedical engineering. Soft Matter.

[92] Audelo M.L.D.P., Mendoza-Muñoz N., Escutia-Guadarrama L., Giraldo-Gomez D.M., González-Torres M., Florán B., Cortes H., Leyva-Gómez G. (2020). Recent advances in elastin-based biomaterials. J. Pharm. Pharm. Sci..

[93] Varanko A.K., Su J.C., Chilkoti A. (2020). Elastin-Like Polypeptides for Biomedical Applications. Annu. Rev. Biomed. Eng..

[94] Unal G., Jones J., Baghdasarian S., Kaneko N., Shirzaei Sani E., Lee S., Gholizadeh S., Tateshima S., Annabi N. (2021). Engineering elastic sealants based on gelatin and elastin-like polypeptides for endovascular anastomosis. Bioeng. Transl. Med..

[95] Tian D.-M., Wan H.-H., Chen J.-R., Ye Y.-B., He Y., Liu Y., Tang L.-Y., He Z.-Y., Liu K.-Z., Gao C.-J. (2022). In-situ formed elastin-based hydrogels enhance wound healing via promoting innate immune cells recruitment and angiogenesis. Mater. Today Bio.

[96] Cipriani F., Krüger M., De Torre I.G., Sierra L.Q., Rodrigo M.A., Kock L., Rodriguez-Cabello J.C. (2018). Cartilage Regeneration in Preannealed Silk Elastin-Like Co-Recombinamers Injectable Hydrogel Embedded with Mature Chondrocytes in an Ex Vivo Culture Platform. Biomacromolecules.

[97] Águila-Almanza E., Low S.S., Hernández-Cocoletzi H., Atonal-Sandoval A., Rubio-Rosas E., Violante-González J., Show P.L. (2021). Facile and green approach in managing sand crab carapace biowaste for obtention of high deacetylation percentage chitosan. J. Environ. Chem. Eng..

[98] Sánchez-Cid P., Jiménez-Rosado M., Alonso-González M., Romero A., Perez-Puyana V. (2021). Applied Rheology as Tool for the Assessment of Chitosan Hydrogels for Regenerative Medicine. Polymers.

[99] Dziadek M., Dziadek K., Salagierski S., Drozdowska M., Serafim A., Stancu I.-C., Szatkowski P., Kopec A., Rajzer I., Douglas T.E.L. (2022). Newly crosslinked chitosan- and chitosan-pectin-based hydrogels with high antioxidant and potential anticancer activity. Carbohydr. Polym..

[100] Fu S., Zhou L., Zeng P., Fu S. (2022). Antibacterial Chitosan-gelatin Hydrogel Beads Cross-linked by Riboflavin under Ultraviolet A Irradiation. Fibers Polym..

[101] Quadrado R.F.N., Macagnan K.L., Moreira A.S., Fajardo A.R. (2021). Chitosan-based hydrogel crosslinked through an aza-Michael addition catalyzed by boric acid. Int. J. Biol. Macromol..

[102] Do N.H.N., Truong Q.T., Le P.K., Ha A.C. (2022). Recent developments in chitosan hydrogels carrying natural bioactive compounds. Carbohydr. Polym..

[103] Liu Z., Wang K., Peng X., Zhang L. (2022). Chitosan-based drug delivery systems: Current strategic design and potential application in human hard tissue repair. Eur. Polym. J..

[104] Maiz-fern S., Silv U. (2022). Photocrosslinkable and self-healable hydrogels of chitosan and hyaluronic acid. Int. J. Biol. Macromol..

[105] Larrañeta E., Henry M., Irwin N.J., Trotter J., Perminova A.A., Donnelly R.F. (2018). Synthesis and characterization of hyaluronic acid hydrogels crosslinked using a solvent-free process for potential biomedical applications. Carbohydr. Polym..

[106] Mihajlovic M., Fermin L., Ito K., Van Nostrum C.F., Vermonden T. (2021). Hyaluronic acid-based supramolecular hydrogels for biomedical applications. Multifunct. Mater..

[107] Pérez L.A., Hernández R., Alonso J.M., Pérez-González R., Sáez-Martínez V. (2021). Hyaluronic acid hydrogels crosslinked in physiological conditions: Synthesis and biomedical applications. Biomedicines.

[108] Salma-Ancane K., Sceglovs A., Tracuma E., Wychowaniec J.K., Aunina K., Ramata-Stunda A., Nikolajeva V., Loca D. (2022). Effect of crosslinking strategy on the biological, antibacterial and physicochemical performance of hyaluronic acid and ɛ-polylysine based hydrogels. Int. J. Biol. Macromol..

[109] Long L., Hu C., Liu W., Wu C., Lu L., Yang L., Wang Y. (2022). Injectable multifunctional hyaluronic acid/methylcellulose hydrogels for chronic wounds repairing. Carbohydr. Polym..

[110] Guo W., Douma L., Hu M.H., Eglin D., Alini M., Šećerović A., Grad S., Peng X., Zou X., D’Este M. (2022). Hyaluronic acid-based interpenetrating network hydrogel as a cell carrier for nucleus pulposus repair. Carbohydr. Polym..

[111] Sciences M. (2022). Application of Alginate Hydrogels for Next-Generation Articular Cartilage Regeneration. Int. J. Mol. Sci..

[112] Zhang H., Cheng J., Ao Q. (2021). Preparation of alginate-based biomaterials and their applications in biomedicine. Mar. Drugs.

[113] Maity C., Das N. (2022). Alginate-Based Smart Materials and Their Application: Recent Advances and Perspectives.

[114] Soleimanpour M., Mirhaji S.S., Jafari S., Derakhshankhah H., Mamashli F., Nedaei H., Karimi M.R., Motasadizadeh H., Fatahi Y., Ghasemi A. (2022). Designing a new alginate-fibrinogen biomaterial composite hydrogel for wound healing. Sci. Rep..

[115] Teixeira M.C., Lameirinhas N.S., Carvalho J.P.F., Valente B.F.A., Luís J., Pires L., Oliveira H., Oliveira M., Silvestre A.J.D., Vilela C. (2022). Alginate-Lysozyme Nanofibers Hydrogels with Improved Rheological Behavior, Printability and Biological Properties for 3D Bioprinting Applications. Nanomaterials.

[116] Hoang H.T., Vu T.T., Karthika V., Jo S.H., Jo Y.J., Seo J.W., Oh C.W., Park S.H., Lim K.T. (2022). Dual cross-linked chitosan/alginate hydrogels prepared by Nb-Tz ‘click’ reaction for pH responsive drug delivery. Carbohydr. Polym..

[117] Zeng Y., Chen C., Liu W., Fu Q., Han Z., Li Y., Feng S., Li X., Qi C., Wu J. (2015). Injectable microcryogels reinforced alginate encapsulation ofmesenchymal stromal cells for leak-proof delivery andalleviationofcanine disc degeneration. Biomaterials.

[118] Bao Y., He J., Song K., Guo J., Zhou X., Liu S. (2022). Functionalization and Antibacterial Applications of Cellulose-Based Composite Hydrogels. Polymers.

[119] Kundu R., Mahada P., Chhirang B., Das B. (2022). Cellulose hydrogels: Green and sustainable soft biomaterials. Curr. Res. Green Sustain. Chem..

[120] Prusty K., Swain S.K. (2021). Polypropylene oxide/polyethylene oxide-cellulose hybrid nanocomposite hydrogels as drug delivery vehicle. J. Appl. Polym. Sci..

[121] Hu Y., Li N., Yue P., Chen G., Hao X., Bian J., Peng F. (2022). Highly antibacterial hydrogels prepared from amino cellulose, dialdehyde xylan, and Ag nanoparticles by a green reduction method. Cellulose.

[122] Eivazzadeh-Keihan R., Khalili F., Khosropour N., Aliabadi H.A.M., Radinekiyan F., Sukhtezari S., Maleki A., Madanchi H., Hamblin M.R., Mahdavi M. (2021). Hybrid Bionanocomposite Containing Magnesium Hydroxide Nanoparticles Embedded in a Carboxymethyl Cellulose Hydrogel plus Silk Fibroin as a Scaffold for Wound Dressing Applications. ACS Appl. Mater. Interfaces.

[123] Song F., Gong J., Tao Y., Cheng Y., Lu J., Wang H. (2021). A robust regenerated cellulose-based dual stimuli-responsive hydrogel as an intelligent switch for controlled drug delivery. Int. J. Biol. Macromol..

[124] Yang G., Zhang Z., Liu K., Ji X., Fatehi P., Chen J. (2022). A cellulose nanofibril—Reinforced hydrogel with robust mechanical, self-healing, pH-responsive and antibacterial characteristics for wound dressing applications. J. Nanobiotechnol..

[125] Pagano C., Calarco P., Di Michele A., Ceccarini M.R., Beccari T., Primavilla S., Scuota S., Marmottini F., Ramella D., Ricci M. (2021). Development of sodium carboxymethyl cellulose based polymeric microparticles for in situ hydrogel wound dressing formation. Int. J. Pharm..

[126] Huang Y.-C., Liu Z.-H., Kuo C.-Y., Chen J.-P. (2022). Photo-Crosslinked Hyaluronic Acid/Carboxymethyl Cellulose Composite Hydrogel as a Dural Substitute to Prevent Post-Surgical Adhesion. Int. J. Mol. Sci..

[127] Xia B., Chen G. (2022). Research progress of natural tissue-derived hydrogels for tissue repair and reconstruction. Int. J. Biol. Macromol..

[128] Hong K.H., Jeon Y.S., Kim J.H. (2009). Preparation and properties of modified PHEMA hydrogels containing thermo-responsive pluronic component. Macromol. Res..

[129] Li R., Guan X., Lin X., Guan P., Zhang X., Rao Z., Du L., Zhao J., Rong J., Zhao J. (2020). Poly(2-hydroxyethyl methacrylate)/β-cyclodextrin-hyaluronan contact lens with tear protein adsorption resistance and sustained drug delivery for ophthalmic diseases. Acta Biomater..

[130] Xie G., Du S., Huang Q., Mo M., Gao Y., Li M., Tao J., Zhang L., Zhu J. (2022). Photonic Hydrogels for Synergistic Visual Bacterial Detection and On-Site Photothermal Disinfection. ACS Appl. Mater. Interfaces.

[131] Buder K., Kaefer K., Flietel B., Uzun H., Schroeder T., Sönnichsen C. (2022). Integrating Nanosensors into Macroporous Hydrogels for Implantation. ACS Appl. Bio Mater..

[132] Bolívar-Monsalve E.J., Alvarez M.M., Hosseini S., Espinosa-Hernandez M.A., Ceballos-González C.F., Sanchez-Dominguez M., Shin S.R., Cecen B., Hassan S., Di Maio E. (2021). Engineering bioactive synthetic polymers for biomedical applications: A review with emphasis on tissue engineering and controlled release. Mater. Adv..

[133] Vales T.P., Jee J.P., Lee W.Y., Min I., Cho S., Kim H.J. (2020). Protein Adsorption and Bacterial Adhesion Resistance of Cross-linked Copolymer Hydrogels Based on Poly(2-methacryloyloxyethyl phosphorylcholine) and Poly(2-hydroxyethyl methacrylate). Bull. Korean Chem. Soc..

[134] Ulu A., Balcioglu S., Birhanli E., Sarimeseli A., Keskin R., Koytepe S., Ates B. (2018). Poly(2-hydroxyethyl methacrylate)/boric acid composite hydrogel as soft contact lens material: Thermal, optical, rheological, and enhanced antibacterial properties. J. Appl. Polym. Sci..

[135] Tomić S.L., Babić M.M., Vuković J.S., Djokić L., Pavić A., Nikodinovic-Runić J. (2020). Effect of composition and method of preparation of 2-hydroxyethyl methacrylate/gelatin hydrogels on biological In Vitro (cell line) and In Vivo (zebrafish) properties. J. Polym. Res..

[136] Chong S.F., Smith A.A.A., Zelikin A.N. (2013). Microstructured, functional PVA hydrogels through bioconjugation with oligopeptides under physiological conditions. Small.

[137] Chopra H., Bibi S., Kumar S., Khan M.S., Kumar P., Singh I. (2022). Preparation and Evaluation of Chitosan/PVA Based Hydrogel Films Loaded with Honey for Wound Healing Application. Gels.

[138] Leone G., Consumi M., Aggravi M., Donati A., Lamponi S., Magnani A. (2010). PVA/STMP based hydrogels as potential substitutes of human vitreous. J. Mater. Sci. Mater. Med..

[139] Stasko J., Kalnińš M., Dzene A., Tupureina V. (2009). Poly(vinyl alcohol) hydrogels. Proc. Est. Acad. Sci..

[140] Ostuni E., Chapman R.G., Holmlin R.E., Takayama S., Whitesides G.M. (2001). A survey of structure-property relationships of surfaces that resist the adsorption of protein. Langmuir.

[141] Lin C.C., Anseth K.S. (2009). PEG hydrogels for the controlled release of biomolecules in regenerative medicine. Pharm. Res..

[142] Naahidi S., Jafari M., Logan M., Wang Y., Yuan Y., Bae H., Dixon B., Chen P. (2017). Biocompatibility of hydrogel-based scaffolds for tissue engineering applications. Biotechnol. Adv..

[143] Cai T., Yang W.J., Zhang Z., Zhu X., Neoh K.G., Kang E.T. (2012). Preparation of stimuli-responsive hydrogel networks with threaded β-cyclodextrin end-capped chains via combination of controlled radical polymerization and click chemistry. Soft Matter.

[144] Lee C.Y., Teymour F., Camastral H., Tirelli N., Hubbell J.A., Elbert D.L., Papavasiliou G. (2014). Characterization of the Network Structure of PEG Diacrylate Hydrogels Formed in the Presence of N-Vinyl Pyrrolidone. Macromol. React. Eng..

[145] Cui H., Shao J., Wang Y., Zhang P., Chen X., Wei Y. (2013). PLA-PEG-PLA and its electroactive tetraaniline copolymer as multi-interactive injectable hydrogels for tissue engineering. Biomacromolecules.

[146] Mann B.K., Gobin A.S., Tsai A.T., Schmedlen R.H., West J.L. (2001). Smooth muscle cell growth in photopolymerized hydrogels with cell adhesive and proteolytically degradable domains: Synthetic ECM analogs for tissue engineering. Biomaterials.

[147] Kumar P., Choonara Y.E. (2021). Thermogelling behaviour of PEG-enclatherated Methylcellulose/Alginate sols. Mater. Res. Express.

[148] Monfared M., Mawad D., Rnjak-Kovacina J., Stenzel M.H. (2021). 3D bioprinting of dual-crosslinked nanocellulose hydrogels for tissue engineering applications. J. Mater. Chem. B.

[149] Lee S.Y., Bang S., Kim S., Jo S.Y., Kim B.C., Hwang Y., Noh I. (2015). Synthesis and In Vitro characterizations of porous carboxymethyl cellulose-poly(ethylene oxide) hydrogel film. Biomater. Res..

[150] Zhang S., Zhou H., Huang C., Sun J., Qu X., Lu Y. (2022). A novel corneal adhesive based on functionally coupled PEG-lysozyme hydrogel for wound closure after surgical eye surgery. Chin. Chem. Lett..

[151] Anufrieva E.V., Gromova R.A., Kirsh Y.E., Yanul N.A., Krakovyak M.G., Lushchik V.B., Pautov V.D., Sheveleva T.V. (2001). Complexing properties and structural characteristics of thermally sensitive copolymers of N-vinylpyrrolidone and N-vinylcaprolactam. Eur. Polym. J..

[152] Mahmood A., Patel D., Hickson B., Desrochers J., Hu X. (2022). Recent Progress in Biopolymer-Based Hydrogel Materials for Biomedical Applications. Int. J. Mol. Sci..

[153] Gough C.R., Callaway K., Spencer E., Leisy K., Jiang G., Yang S., Hu X. (2021). Biopolymer-based filtration materials. ACS Omega.

[154] Pattanashetti N.A., Heggannavar G.B., Kariduraganavar M.Y. (2017). Smart Biopolymers and their Biomedical Applications. Procedia Manuf..

[155] Hutmacher D.W. (2000). Scaffolds in tissue engineering bone and cartilage. Biomater. Silver Jubil. Compend..

[156] Saroia J., Yanen W., Wei Q., Zhang K., Lu T., Zhang B. (2018). A review on biocompatibility nature of hydrogels with 3D printing techniques, tissue engineering application and its future prospective. Bio-Des. Manuf..

[157] Gough C.R., Rivera-Galletti A., Cowan D.A., Salas-De La Cruz D., Hu X. (2020). Protein and polysaccharide-based fiber materials generated from ionic liquids: A review. Molecules.

[158] Alphonsa A., Hakkim S., Lakshmi N.M., Arun K.B., Madhavan A., Sirohi R., Tarafdar A., Sindhu R., Kumar M., Pandey A. (2022). Bacterial biopolymers: From production to applications in biomedicine. Sustain. Chem. Pharm..

[159] Xu X., Xu Z.K., Yang X.F., He Y.H., Lin R. (2017). Construction and characterization of a pure protein hydrogel for drug delivery application. Int. J. Biol. Macromol..

[160] Ahn W., Lee J.H., Kim S.R., Lee J., Lee E.J. (2021). Designed protein- And peptide-based hydrogels for biomedical sciences. J. Mater. Chem. B.

[161] Tang Y., Zhang X., Li X., Ma C., Chu X., Wang L., Xu W. (2022). A review on recent advances of Protein-Polymer hydrogels. Eur. Polym. J..

[162] Davari N., Bakhtiary N., Khajehmohammadi M., Sarkari S., Tolabi H., Ghorbani F., Ghalandari B. (2022). Protein-Based Hydrogels: Promising Materials for Tissue Engineering. Polymers.

[163] Kasoju N., Ali S.S., Dubey V.K., Bora U. (2010). Exploiting the potential of collagen as a natural biomaterial in drug delivery. J. Proteins Proteomics.

[164] Bolke L., Schlippe G., Gerß J., Voss W. (2019). A collagen supplement improves skin hydration, elasticity, roughness, and density: Results of a randomized, placebo-controlled, blind study. Nutrients.

[165] Sherman V.R., Yang W., Meyers M.A. (2015). The materials science of collagen. J. Mech. Behav. Biomed. Mater..

[166] Jaipan P., Nguyen A., Narayan R.J. (2017). Gelatin-based hydrogels for biomedical applications. MRS Commun..

[167] Sethi S., Singh G., Sharma R., Kaith B.S., Sharma N., Khullar S. (2022). Fluorescent hydrogel of chitosan and gelatin cross-linked with maleic acid for optical detection of heavy metals. J. Appl. Polym. Sci..

[168] Dou C., Li Z., Luo Y., Gong J., Li Q., Zhang J., Zhang Q., Qiao C. (2022). Bio-based poly (γ-glutamic acid)-gelatin double-network hydrogel with high strength for wound healing. Int. J. Biol. Macromol..

[169] Chen H., Wu D., Ma W., Wu C., Liu J., Du M. (2022). Strong fish gelatin hydrogels double crosslinked by transglutaminase and carrageenan. Food Chem..

[170] Ye J., Xiao Z., Gao L., Zhang J., He L., Zhang H., Liu Q., Yang G. (2021). Assessment of the effects of four crosslinking agents on gelatin hydrogel for myocardial tissue engineering applications. Biomed. Mater..

[171] Sakr M.A., Sakthivel K., Hossain T., Shin S.R., Siddiqua S., Kim J., Kim K. (2022). Recent trends in gelatin methacryloyl nanocomposite hydrogels for tissue engineering. J. Biomed. Mater. Res.-Part A.

[172] Lee Y.S., Bae J.Y., Koo H.Y., Lee Y.B., Choi W.S. (2016). A remote-controlled generation of gold@polydopamine (core@shell) nanoparticles via physical-chemical stimuli of polydopamine/gold composites. Sci. Rep..

[173] Sell S.A., McClure M.J., Barnes C.P., Knapp D.C., Walpoth B.H., Simpson D.G., Bowlin G.L. (2006). Electrospun polydioxanone-elastin blends: Potential for bioresorbable vascular grafts. Biomed. Mater..

[174] Almine J.F., Bax D.V., Mithieux S.M., Smith L.N., Rnjak J., Waterhouse A., Wise S.G., Weiss A.S. (2010). Elastin-based materials. Chem. Soc. Rev..

[175] Kyle S., Aggeli A., Ingham E., McPherson M.J. (2009). Production of self-assembling biomaterials for tissue engineering. Trends Biotechnol..

[176] Alavarse A.C., Frachini E.C.G., da Silva R.L.C.G., Lima V.H., Shavandi A., Petri D.F.S. (2022). Crosslinkers for polysaccharides and proteins: Synthesis conditions, mechanisms, and crosslinking efficiency, a review. Int. J. Biol. Macromol..

[177] Guo C., Zeng Z., Yu S., Huang J., Geng Z., Pei D., Lu D. (2022). Synthesis of bovine serum albumin-gelatin composite adhesive hydrogels by physical crosslinking. J. Polym. Res..

[178] Ye W., Qin M., Qiu R., Li J. (2022). Keratin-based wound dressings: From waste to wealth. Int. J. Biol. Macromol..

[179] Pepe A., Maio L., Bracalello A., Quintanilla-Sierra L., Arias F.J., Girotti A., Bochicchio B. (2021). Soft Hydrogel Inspired by Elastomeric Proteins. ACS Biomater. Sci. Eng..

[180] Mei J., Zhou J., Kong L., Dai Y., Zhang X., Song W., Zhu C. (2022). An injectable photo-cross-linking silk hydrogel system augments diabetic wound healing in orthopaedic surgery through spatiotemporal immunomodulation. J. Nanobiotechnol..

[181] Lovegrove A., Edwards C.H., De Noni I., Patel H., El S.N., Grassby T., Zielke C., Ulmius M., Nilsson L., Butterworth P.J. (2017). Role of polysaccharides in food, digestion, and health. Crit. Rev. Food Sci. Nutr..

[182] Khodadadi M., Zarrintaj P., Khodadadi A., Arefi A., Seidi F., Shokrani H., Reza M., Mozafari M. (2022). Polysaccharide-based electroconductive hydrogels: Structure, properties and biomedical applications. Carbohydr. Polym..

[183] Seidi F., Zhao W.F., Xiao H.N., Jin Y.C., Saeb M.R., Zhao C.S. (2021). Advanced Surfaces by Anchoring Thin Hydrogel Layers of Functional Polymers. Chin. J. Polym. Sci..

[184] Khattab T.A., Kamel S. (2022). Advances in polysaccharide-based hydrogels: Self-healing and electrical conductivity. J. Mol. Liq..

[185] Fragal E.H., Fragal V.H., Silva E.P., Paulino A.T., da Silva Filho E.C., Mauricio M.R., Silva R., Rubira A.F., Muniz E.C. (2022). Magnetic-responsive polysaccharide hydrogels as smart biomaterials: Synthesis, properties, and biomedical applications. Carbohydr. Polym..

[186] Raafat D., Sahl H.G. (2009). Chitosan and its antimicrobial potential—A critical literature survey. Microb. Biotechnol..

[187] Khan A., Alamry K.A., Asiri A.M. (2021). Multifunctional Biopolymers-Based Composite Materials for Biomedical Applications: A Systematic Review. ChemistrySelect.

[188] Liu Y., Lin S.-H., Chuang W.-T., Dai N.-T., Hsu S. (2022). Biomimetic Strain-Stiffening in Chitosan Self-Healing Hydrogels. ACS Appl. Mater. Interfaces.

[189] Sánchez-Cid P., Jiménez-Rosado M., Rubio-Valle J.F., Romero A., Ostos F.J., Benhnia R.E.I., Perez-Puyana V. (2022). Biocompatible and Thermoresistant Hydrogels Based on Collagen and Chitosan. Polymers.

[190] Michailidou G., Koukaras E.N., Bikiaris D.N. (2021). Vanillin chitosan miscible hydrogel blends and their prospects for 3D printing biomedical applications. Int. J. Biol. Macromol..

[191] Fourie J., Taute F., du Preez L., de Beer D. (2021). Novel chitosan-poly(vinyl acetate) biomaterial suitable for additive manufacturing and bone tissue engineering applications. J. Bioact. Compat. Polym..

[192] Bazghaleh A.A., Dogolsar M.A., Barzin J. (2022). Development of an injectable self-healing hydrogel based on N-succinyl chitosan/ oxidized pectin for biomedical applications. J. Polym. Res..

[193] Jiménez-Gómez C.P., Cecilia J.A. (2020). Chitosan: A Natural Biopolymer with a Wide and Varied Range of Applications. Molecules.

[194] Kharkar P.M., Kiick K.L., Kloxin A.M. (2013). Designing degradable hydrogels for orthogonal control of cell microenvironments. Chem. Soc. Rev..

[195] Ahmad Raus R., Wan Nawawi W.M.F., Nasaruddin R.R. (2021). Alginate and alginate composites for biomedical applications. Asian J. Pharm. Sci..

[196] Camponeschi F., Atrei A., Rocchigiani G., Mencuccini L., Uva M., Barbucci R. (2015). New formulations of polysaccharide-based hydrogels for drug release and tissue engineering. Gels.

[197] Zhang X.N., Zheng Q., Wu Z.L. (2022). Recent advances in 3D printing of tough hydrogels: A review. Compos. Part B Eng..

[198] Correa S., Grosskopf A.K., Lopez Hernandez H., Chan D., Yu A.C., Stapleton L.M., Appel E.A. (2021). Translational Applications of Hydrogels. Chem. Rev..

[199] Vareda J.P., Lamy-Mendes A., Durães L. (2018). A reconsideration on the definition of the term aerogel based on current drying trends. Microporous Mesoporous Mater..

[200] Lai J.Y., Luo L.J., Ma D.H.K. (2018). Effect of cross-linking density on the structures and properties of carbodiimide-treated gelatin matrices as limbal stem cell niches. Int. J. Mol. Sci..

[201] Balcioglu S., Gurses C., Ozcan I., Yildiz A., Koytepe S., Parlakpinar H., Vardi N., Ates B. (2021). Photocrosslinkable gelatin/collagen based bioinspired polyurethane-acrylate bone adhesives with biocompatibility and biodegradability. Int. J. Biol. Macromol..

[202] Yao G., Liu X., Zhang G., Han Z., Liu H. (2021). Green synthesis of tannic acid functionalized graphene hydrogel to efficiently adsorb methylene blue. Colloids Surfaces A Physicochem. Eng. Asp..

[203] Nahar Y., Thickett S.C. (2021). Greener, faster, stronger: The benefits of deep eutectic solvents in polymer and materials science. Polymers.

[204] Qureshi M.A., Nishat N., Jadoun S., Ansari M.Z. (2020). Polysaccharide based superabsorbent hydrogels and their methods of synthesis: A review. Carbohydr. Polym. Technol. Appl..

[205] Kesharwani P., Bisht A., Alexander A., Dave V., Sharma S. (2021). Biomedical applications of hydrogels in drug delivery system: An update. J. Drug Deliv. Sci. Technol..

[206] Akhtar M.F., Hanif M., Ranjha N.M. (2016). Methods of synthesis of hydrogels … A review. Saudi Pharm. J..

[207] Man Z., Sidi L., Xubo Y., Jin Z., Xin H. (2021). An in situ catechol functionalized ε-polylysine/polyacrylamide hydrogel formed by hydrogen bonding recombination with high mechanical property for hemostasis. Int. J. Biol. Macromol..

[208] Jing H., Huang X., Du X., Mo L., Ma C., Wang H. (2022). Facile synthesis of pH-responsive sodium alginate/carboxymethyl chitosan hydrogel beads promoted by hydrogen bond. Carbohydr. Polym..

[209] Castillo R.V., Müller A.J. (2009). Crystallization and morphology of biodegradable or biostable single and double crystalline block copolymers. Prog. Polym. Sci..

[210] Cheol Kim H., Na Lee J., Kim E., Hee Kim M., Ho Park W. (2021). Self-healable poly(γ-glutamic acid)/chitooligosaccharide hydrogels via ionic and π-interactions. Mater. Lett..

[211] Yuan N., Xu L., Xu B., Zhao J., Rong J. (2018). Chitosan derivative-based self-healable hydrogels with enhanced mechanical properties by high-density dynamic ionic interactions. Carbohydr. Polym..

[212] Ullah A., Lim S.I. (2022). Bioinspired tunable hydrogels: An update on methods of preparation, classification, and biomedical and therapeutic applications. Int. J. Pharm..

[213] Ribotta P.D., Colombo A., Rosell C.M. (2012). Enzymatic modifications of pea protein and its application in protein–cassava and corn starch gels. Food Hydrocoll..

[214] Gaar J., Naffa R., Brimble M. (2020). Enzymatic and non-enzymatic crosslinks found in collagen and elastin and their chemical synthesis. Org. Chem. Front..

[215] Perez-Puyana V., Jiménez-Rosado M., Romero A., Guerrero A. (2019). Crosslinking of hybrid scaffolds produced from collagen and chitosan. Int. J. Biol. Macromol..

[216] Wolfel A., Romero M.R., Alvarez Igarzabal C.I. (2017). Post-synthesis modification of hydrogels. Total and partial rupture of crosslinks: Formation of aldehyde groups and re-crosslinking of cleaved hydrogels. Polymer.

[217] Nezhad-Mokhtari P., Ghorbani M., Roshangar L., Soleimani Rad J. (2019). Chemical gelling of hydrogels-based biological macromolecules for tissue engineering: Photo- and enzymatic-crosslinking methods. Int. J. Biol. Macromol..

[218] Shahi S., Roghani-Mamaqani H., Talebi S., Mardani H. (2022). Stimuli-responsive destructible polymeric hydrogels based on irreversible covalent bond dissociation. Polym. Chem..

[219] Zhong Y., Wang J., Yuan Z., Wang Y., Xi Z., Li L., Liu Z., Guo X. (2019). A mussel-inspired carboxymethyl cellulose hydrogel with enhanced adhesiveness through enzymatic crosslinking. Colloids Surfaces B Biointerfaces.

[220] Campea M.A., Majcher M.J., Lofts A., Hoare T. (2021). A Review of Design and Fabrication Methods for Nanoparticle Network Hydrogels for Biomedical, Environmental, and Industrial Applications. Adv. Funct. Mater..

[221] Liu Y., Wei H., Li S., Wang G., Guo T., Han H. (2022). Facile fabrication of semi-IPN hydrogel adsorbent based on quaternary cellulose via amino-anhydride click reaction in water. Int. J. Biol. Macromol..

[222] Bhattacharjee P., Ahearne M. (2021). Significance of crosslinking approaches in the development of next generation hydrogels for corneal tissue engineering. Pharmaceutics.

[223] Patil S., Jadge D. (2016). Crosslinking of Polysaccharides: Methods And Applications Crosslinking Of Polysaccharides: Methods And Applications. Pharm. Rev..

[224] Feng H., Tian C., Zhang G., Zhang L. (2021). Catalyst-free curing and closed-loop recycling of carboxylated functionalized rubber by a green crosslinking strategy. Polymer.

[225] Correa R.F., Colucci G., Halla N., Santamaria-echart A., Blanco S.P., Patr I., Barreiro M.F. (2021). Development of Chitosan Microspheres through a Green Dual. Molecules.

[226] Li C., Wang L., Chen Z., Li Y., Li J. (2020). Facile and green preparation of diverse arabinoxylan hydrogels from wheat bran by combining subcritical water and enzymatic crosslinking. Carbohydr. Polym..

[227] Ghavaminejad A., Ashammakhi N., Wu X.Y., Khademhosseini A. (2020). Crosslinking Strategies for Three-Dimensional Bioprinting of Polymeric Hydrogels. Small.

[228] Vasile C., Pamfil D., Stoleru E., Baican M. (2020). New developments in medical applications of hybrid hydrogels containing natural polymers. Molecules.

[229] Zeimaran E., Pourshahrestani S., Fathi A., Anuar N., Adib N., Sheikhi A., Baino F. (2021). Acta Biomaterialia Advances in bioactive glass-containing injectable hydrogel biomaterials for tissue regeneration. Acta Biomater..

[230] Biondi M., Borzacchiello A., Mayol L., Ambrosio L. (2015). Nanoparticle-integrated hydrogels as multifunctional composite materials for biomedical applications. Gels.

[231] Hasan M.S., Al Foisal J., Khan G.M.A., Jahan R., Hasanuzzaman M., Alam M.S., Karim M.M., Gafur M.A., Khan M.A., Sabur M.A. (2022). Microfibrillated Cellulose-Silver Nanocomposite Based PVA Hydrogels and Their Enhanced Physical, Mechanical and Antibacterial Properties. J. Polym. Environ..

[232] Kozicki M., Pawlaczyk A., Adamska A., Iwona M. (2022). Golden and Silver—Golden Chitosan Hydrogels and Fabrics Modified with Golden Chitosan Hydrogels. Int. J. Mol. Sci..

[233] Zeng Q., Wu T. (2022). Enhanced electrochemical performance of neural electrodes based on PEDOT:PSS hydrogel. J. Appl. Polym. Sci..

[234] Boztepe C., Daskin M., Erdogan A., Sarici T. (2021). Preparation of poly(acrylamide-co-2-acrylamido-2-methylpropan sulfonic acid)-g-Carboxymethyl cellulose/Titanium dioxide hydrogels and modeling of their swelling capacity and mechanic strength behaviors by response surface method technique. Polym. Eng. Sci..

[235] Bustamante-Torres M., Romero-Fierro D., Estrella-Nuñez J., Arcentales-Vera B., Chichande-Proaño E., Bucio E. (2022). Polymeric Composite of Magnetite Iron Oxide Nanoparticles and Their Application in Biomedicine: A Review. Polymers.

[236] Ghanbari M., Salavati-Niasari M., Mohandes F., Firouzi Z., Mousavi S.D. (2021). The impact of zirconium oxide nanoparticles content on alginate dialdehyde-gelatin scaffolds in cartilage tissue engineering. J. Mol. Liq..

[237] Zhang W., Huang G., Ng K., Ji Y., Gao B., Huang L., Zhou J., Lu T.J., Xu F. (2018). Engineering ellipsoidal cap-like hydrogel particles as building blocks or sacrificial templates for three-dimensional cell culture. Biomater. Sci..

[238] Yang C.M., Lee J., Lee H., Park W.H. (2022). ZnO nanoparticle-embedded modified silk fibroin-tannin multifunctional hydrogel. Int. J. Biol. Macromol..

[239] Tan H.L., Teow S.Y., Pushpamalar J. (2019). Application of metal nanoparticle–hydrogel composites in tissue regeneration. Bioengineering.

[240] Thoniyot P., Tan M.J., Karim A.A., Young D.J., Loh X.J. (2015). Nanoparticle–Hydrogel Composites: Concept, Design, and Applications of These Promising, Multi-Functional Materials. Adv. Sci..

[241] Gaharwar A.K., Peppas N.A., Khademhosseini A. (2014). Nanocomposite hydrogels for biomedical applications. Biotechnol. Bioeng..

[242] Filipović V.V., Babić Radić M.M., Vuković J.S., Vukomanović M., Rubert M., Hofmann S., Müller R., Tomić S.L. (2021). Biodegradable Hydrogel Scaffolds Based on 2-Hydroxyethyl. Polymers.

[243] Yoshikawa H., Myoui A. (2005). Bone tissue engineering with porous hydroxyapatite ceramics. J. Artif. Organs.

[244] Zhao R., Xie P., Zhang K., Tang Z., Chen X., Zhu X., Fan Y., Yang X., Zhang X. (2017). Selective effect of hydroxyapatite nanoparticles on osteoporotic and healthy bone formation correlates with intracellular calcium homeostasis regulation. Acta Biomater..

[245] Mohamed Haneef I.N.H., Mohd Shaffiar N., Buys Y.F., Syed Shaharuddin S.I., Abdul Hamid A.M., Widiyati K. (2022). Recent advancement in polymer/halloysite nanotube nanocomposites for biomedical applications. J. Biomed. Mater. Res.-Part B Appl. Biomater..

[246] Huang K., Ou Q., Xie Y., Chen X., Fang Y., Huang C., Wang Y., Gu Z., Wu J. (2019). Halloysite Nanotube Based Scaffold for Enhanced Bone Regeneration. ACS Biomater. Sci. Eng..

[247] Wong L.W., Goh C.B.S., Pasbakhsh P., Tan J.B.L. (2022). Natural hollow clay nanotubes and their applications as polymer nanocomposites in tissue engineering. J. Sci. Adv. Mater. Devices.

[248] Naumenko E., Guryanov I., Zakirova E., Fakhrullin R. (2021). Forskolin-loaded halloysite nanotubes as osteoconductive additive for the biopolymer tissue engineering scaffolds. Polymers.

[249] dos Santos L., Malmonge S.M., Santos L.R., Rodas A.C.D., Daguano J.K.M.B. (2021). Processing and properties of a Chitosan-Hyaluronic Acid-Biosilicate ^®^ (CHI-HA-BioS) composite for wound healing applications. Res. Biomed. Eng..

[250] Maleki A., He J., Bochani S., Nosrati V., Shahbazi M.A., Guo B. (2021). Multifunctional Photoactive Hydrogels for Wound Healing Acceleration. ACS Nano.

[251] Mellati A., Hasanzadeh E., Gholipourmalekabadi M., Enderami S.E. (2021). Injectable nanocomposite hydrogels as an emerging platform for biomedical applications: A review. Mater. Sci. Eng. C.

[252] Maiti D., Tong X., Mou X., Yang K. (2019). Carbon-Based Nanomaterials for Biomedical Applications: A Recent Study. Front. Pharmacol..

[253] Guilbaud-Chéreau C., Wagner L., Dinesh B., Bianco A., Chaloin O., Ménard-Moyon C. (2022). Aromatic Dipeptide Homologue-Based Hydrogels for Photocontrolled Drug Release. Nanomaterials.

[254] Park S.Y., Kang J.H., Kim H.S., Hwang J.Y., Shin U.S. (2022). Electrical and thermal stimulus-responsive nanocarbon-based 3D hydrogel sponge for switchable drug delivery. Nanoscale.

[255] Kim Y., Song J., Park S.C., Ahn M., Park M.J., Song S.H., Yoo S.Y., Hong S.G., Hong B.H. (2021). Photoinitiated polymerization of hydrogels by graphene quantum dots. Nanomaterials.

[256] Mamidi N., Gamero M.R.M., Castrejón J.V., Zúníga A.E. (2019). Development of ultra-high molecular weight polyethylene-functionalized carbon nano-onions composites for biomedical applications. Diam. Relat. Mater..

[257] Mamidi N., Villela Castrejón J., González-Ortiz A. (2020). Rational design and engineering of carbon nano-onions reinforced natural protein nanocomposite hydrogels for biomedical applications. J. Mech. Behav. Biomed. Mater..

[258] Mamidi N., Velasco Delgadillo R.M., Barrera E.V. (2021). Covalently Functionalized Carbon Nano-Onions Integrated Gelatin Methacryloyl Nanocomposite Hydrogel Containing γ-Cyclodextrin as Drug Carrier for High-Performance pH-Triggered Drug Release. Pharmaceuticals.

[259] Dhand A.P., Galarraga J.H., Burdick J.A. (2021). Enhancing Biopolymer Hydrogel Functionality through Interpenetrating Networks. Trends Biotechnol..

[260] Dragan E.S. (2014). Design and applications of interpenetrating polymer network hydrogels. A review. Chem. Eng. J..

[261] Pal D., Nayak A.K., Saha S. (2019). Interpenetrating Polymer Network Hydrogels of Chitosan: Applications in Controlling Drug Release. Cellulose-Based Superabsorbent Hydrogels.

[262] Dragan E.S., Dinu M.V. (2020). Advances in porous chitosan-based composite hydrogels: Synthesis and applications. React. Funct. Polym..

[263] Lopéz-Martínez E.E., Claudio-Rizo J.A., Caldera-Villalobos M., Becerra-Rodríguez J.J., Cabrera-Munguía D.A., Cano-Salazar L.F., Betancourt-Galindo R. (2022). Hydrogels for Biomedicine Based on Semi-Interpenetrating Polymeric Networks of Collagen/Guar Gum: Synthesis and Physicochemical Characterization. Macromol. Res..

[264] León-Campos M.I., Claudio-Rizo J.A., Rodriguez-Fuentes N., Cabrera-Munguía D.A., Becerra-Rodriguez J.J., Herrera-Guerrero A., Soriano-Corral F. (2021). Biocompatible interpenetrating polymeric networks in hydrogel state comprised from jellyfish collagen and polyurethane. J. Polym. Res..

[265] Claudio-Rizo J.A., Hernandez-Hernandez N.G., Cano-Salazar L.F., Flores-Guía T.E., de la Cruz-Durán F.N., Cabrera-Munguía D.A., Becerra-Rodríguez J.J. (2021). Novel semi-interpenetrated networks based on collagen-polyurethane-polysaccharides in hydrogel state for biomedical applications. J. Appl. Polym. Sci..

[266] Ugrinovic V., Panic V., Spasojevic P., Seslija S., Bozic B., Petrovic R., Janackovic D., Veljovic D. (2022). Strong and tough, pH sensible, interpenetrating network hydrogels based on gelatin and poly(methacrylic acid). Polym. Eng. Sci..

[267] Chen Q., Tian X., Fan J., Tong H., Ao Q. (2020). An Interpenetrating Alginate/Gelatin Network for Three-Dimensional (3D) Cell Cultures and Organ Bioprinting. Molecules.

[268] Hago E.E., Li X. (2013). Interpenetrating polymer network hydrogels based on gelatin and PVA by biocompatible approaches: Synthesis and characterization. Adv. Mater. Sci. Eng..

[269] Aparicio-Collado J.L., García-San-Martín N., Molina-Mateo J., Torregrosa Cabanilles C., Donderis Quiles V., Serrano-Aroca A., Sabater i Serra R. (2022). Electroactive calcium-alginate/polycaprolactone/reduced graphene oxide nanohybrid hydrogels for skeletal muscle tissue engineering. Colloids Surfaces B Biointerfaces.

[270] Claudio-Rizo J.A., Escobedo-Estrada N., Carrillo-Cortes S.L., Cabrera-Munguía D.A., Flores-Guía T.E., Becerra-Rodriguez J.J. (2021). Highly absorbent hydrogels comprised from interpenetrated networks of alginate–polyurethane for biomedical applications. J. Mater. Sci. Mater. Med..

[271] García-Verdugo K.F., Tánori-Córdova J., Rodríguez-Félix F., Ledezma-Pérez A., Castillo-castro T. (2022). A pH/Temperature-Sensitive s-IPN Based on Poly (vinyl alcohol), Poly (vinyl methyl ether- alt -maleic acid) and Poly (vinyl methyl ether) Prepared by Autoclaving. Macromol. Res..

[272] Su Z.C., Lin S.J., Chang Y.H., Yeh W.L., Chu I.M. (2020). Synthesis, characterization, and cytotoxicity of PCL–PEG–PCL diacrylate and agarose interpenetrating network hydrogels for cartilage tissue engineering. J. Appl. Polym. Sci..

[273] O’Brien S., Brannigan R.P., Ibanez R., Wu B., O’Dwyer J., O’Brien F.J., Cryan S.A., Heise A. (2020). Biocompatible polypeptide-based interpenetrating network (IPN) hydrogels with enhanced mechanical properties. J. Mater. Chem. B.

[274] Adelnia H., Blakey I., Little P.J., Ta H.T. (2019). Hydrogels Based on Poly(aspartic acid): Synthesis and Applications. Front. Chem..

[275] James J., Thomas G.V., Akhina H., Thomas S. (2016). Micro-and Nano-Structured Interpenetrating Polymer Networks: State of the Art, New Challenges and Opportunities. Micro- and Nano-Structured Interpenetrating Polymer Networks: From Design to Applications.

[276] Cui C., Jia Y., Sun Q., Yu M., Ji N., Dai L., Wang Y., Qin Y., Xiong L., Sun Q. (2022). Recent advances in the preparation, characterization, and food application of starch-based hydrogels. Carbohydr. Polym..

[277] Ashok Sharma S.S., Bashir S., Kasi R., Subramaniam R.T. (2022). The significance of graphene based composite hydrogels as smart materials: A review on the fabrication, properties, and its applications. FlatChem.

[278] Mamidi N., Delgadillo R.M.V. (2021). Design, fabrication and drug release potential of dual stimuli-responsive composite hydrogel nanoparticle interfaces. Colloids Surfaces B Biointerfaces.

[279] Li Z., Liu L., Chen Y. (2022). Direct 3D printing of thermosensitive AOP127-oxidized dextran hydrogel with dual dynamic crosslinking and high toughness. Carbohydr. Polym..

[280] Hu T., Cui X., Zhu M., Wu M., Tian Y., Yao B., Song W., Niu Z., Huang S., Fu X. (2020). 3D-printable supramolecular hydrogels with shear-thinning property: Fabricating strength tunable bioink via dual crosslinking. Bioact. Mater..

[281] Jain P., Kathuria H., Dubey N. (2022). Advances in 3D bioprinting of tissues/organs for regenerative medicine and in-vitro models. Biomaterials.

[282] Wang Y., Yuan X., Yao B., Zhu S., Zhu P., Huang S. (2022). Tailoring bioinks of extrusion-based bioprinting for cutaneous wound healing. Bioact. Mater..

[283] Zhang Y., Huang Y. (2021). Rational Design of Smart Hydrogels for Biomedical Applications. Front. Chem..

[284] Friuli M., Cafarchia C., Lia R.P., Otranto D., Pombi M., Demitri C. (2022). From tissue engineering to mosquitoes: Biopolymers as tools for developing a novel biomimetic approach to pest management/vector control. Parasites Vectors.

